# Pentaminomycins C–E: Cyclic Pentapeptides as Autophagy Inducers from a Mealworm Beetle Gut Bacterium

**DOI:** 10.3390/microorganisms8091390

**Published:** 2020-09-10

**Authors:** Sunghoon Hwang, Ly Thi Huong Luu Le, Shin-Il Jo, Jongheon Shin, Min Jae Lee, Dong-Chan Oh

**Affiliations:** 1 Natural Products Research Institute, College of Pharmacy, Seoul National University, 1 Gwanak-ro, Gwanak-gu, Seoul 08826, Korea; sunghooi@snu.ac.kr (S.H.); shinj@snu.ac.kr (J.S.); 2 Department of Biochemistry and Molecular Biology, College of Medicine, Seoul National University, Seoul 03080, Korea; huongluuly94@snu.ac.kr; 3 Welfare Division, Seoul Zoo, Seoul Grand Park, Gwacheon, Gyeonggi 13829, Korea; metather@seoul.go.kr

**Keywords:** insect, mealworm, gut bacteria, OSMAC, cyclic peptides, biosynthetic pathway, autophagy inducer

## Abstract

Pentaminomycins C–E (**1**–**3**) were isolated from the culture of the *Streptomyces* sp. GG23 strain from the guts of the mealworm beetle, *Tenebrio molitor*. The structures of the pentaminomycins were determined to be cyclic pentapeptides containing a modified amino acid, *N*^5^-hydroxyarginine, based on 1D and 2D NMR and mass spectroscopic analyses. The absolute configurations of the amino acid residues were assigned using Marfey’s method and bioinformatics analysis of their nonribosomal peptide biosynthetic gene cluster (BGC). Detailed analysis of the BGC enabled us to propose that the structural variations in **1–3** originate from the low specificity of the adenylation domain in the nonribosomal peptide synthetase (NRPS) module 1, and indicate that macrocyclization can be catalyzed noncanonically by penicillin binding protein (PBP)-type TE. Furthermore, pentaminomycins C and D (**1** and **2**) showed significant autophagy-inducing activities and were cytoprotective against oxidative stress in vitro.

## 1. Introduction

Microbial secondary metabolites have been utilized for the discovery and development of innumerable medicinal drugs, such as antibiotics, anticancer agents, and immunosuppressive medicines [1]. However, as more microbial compounds have been reported, the discovery of structurally unique and biologically active compounds has become more difficult. Thus, the development and application of new technologies in exploring new or less-investigated metabolites is particularly important in the field of microbial natural product chemistry. One possible approach is the chemical examination of relatively unexplored microbes [2]. For example, insect-associated bacteria, which are expected to play various roles in the life cycles of hosts due to their range of secondary metabolites, are one of the most unexplored chemical sources [3]. In particular, insects are assumed to actively associate with chemically prolific actinobacteria, which generally originate from the soil. Indeed, studying the metabolites of insect-associated actinomycetes led to the discovery of the new cyclic depsipeptide dentigerumycin [4], a geldanamycin analog natalamycin [5], the polyketide alkaloid camporidine A [6], *N*-acetylcysteamine-bearing indanone thioester formicin A [7], and chlorinated cyclic peptides, nicrophorusamides [8]. These bacterial secondary metabolites display antifungal, anti-inflammatory, anticancer, and antibacterial activities. In addition, *Streptomyces* spp. of the dung beetle *Copris tripartius* have also demonstrated the diversity of bioactive secondary metabolites of insect-associated bacteria, including a tricyclic lactam [9,10], a dichlorinated indanone [11], 2-alkenyl cinnamic acid-bearing cyclic peptides [12], and naphthoquinone-oxindoles [13].

Besides focusing on relatively understudied microbes, another method to efficiently discover new bioactive secondary metabolites is to diversify the culture conditions of one strain, which may induce the biosynthesis of various microbial metabolites that have not been produced under a previous set of conditions. This one strain many compounds (OSMAC) approach [14] is based on the fact that diverse biosynthetic gene clusters exist in the genome of a single strain.

**Figure 1:**
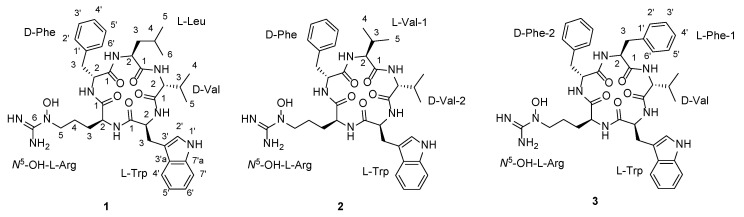
Structures of pentaminomycins C–E (**1**–**3**).

Inspired by these two approaches for the efficient discovery of new bioactive metabolites, we collected mealworm beetles, *Tenebrio molitor*, and isolated actinobacteria from the guts. Initial chemical profiling and subsequent chemical analysis of *Streptomyces* sp. GG23 identified the production of lydiamycin A [15]. Detailed structure elucidation enabled us to revise the previously reported structure [16]. Analysis of the full genome of *Streptomyces* sp. GG23 disclosed its chemical capacity due to the fact that it possesses 31 biosynthetic gene clusters for various structural classes of secondary metabolites in its 8.7 Mb genome (Table S2). Therefore, we altered the culture conditions to induce the production of bacterial metabolites other than lydiamycin. As a result of diverse changes in the composition of the culture media, the production of a series of structurally distinct peptides, namely pentaminomycins C–E (**1**–**3**) (Figure 1), which were barely detected by LC-ESI-MS as minor metabolites when lydiamycin A was produced, was significantly increased. Pentaminomycins A and B, which were produced by *Streptomyces* sp. from Japanese soil have been previously reported as inhibitors of melanin synthesis [17], while pentaminomycin C, which was produced by *Streptomyces cacaoi* isolated from Nigerian cacao beans, has been identified as an antibiotic against Gram-positive bacteria [18]. Thus, we herein report our investigation into the evaluation of pentaminomycins C–E (**1**–**3**) as autophagy inducers and antagonizers against menadione-induced oxidative stress, which have not been previously reported for pentaminomycins. Overall, we report the structures, biological activities, and biosynthetic pathways of pentaminomycins C–E.

## 2. Materials and Methods

### 2.1. General Experimental Procedures

Optical rotations were measured using a JASCO P-2000 polarimeter (JASCO, Easton, MD, USA). UV spectra were recorded using a Chirascan Plus Applied Photophysics Ltd. (Applied Photophysics, Leatherhead, Surrey, UK). Infrared (IR) spectra were obtained on a Thermo NICOLET iS10 spectrometer (Thermo Fisher Scientific, Waltham, MA, USA). ^1^H, ^13^C, and two dimensional nuclear magnetic resonance (NMR) spectra were acquired using a Bruker Avance 800 MHz spectrometer (Bruker, Billerica, MA, USA) with the NMR solvent of DMSO-*d*_6_ (reference chemical shifts: δ_C_ 39.5 ppm and δ_H_ 2.50 ppm) at the National Center for Inter-university Research Facilities (NCIRF) at Seoul National University. Electrospray ionization (ESI) low-resolution liquid chromatography-mass spectrometry (LC/MS)data were measured with an Agilent Technologies 6130 quadrupole mass spectrometer (Agilent, Santa Clara, CA, USA) coupled to an Agilent Technologies 1200 series high-performance liquid chromatography (HPLC) instrument using a reversed-phase C_18_(2) column (Phenomenex Luna, 100 × 4.6 mm). High-resolution fast atom bombardment (HR-FAB) mass spectra were recorded using a Jeol JMS-600 W high-resolution mass spectrometer (JEOL, Akishima, Tokyo, Japan) at the NCIRF. Semi-preparative HPLC separations were achieved using a Gilson 305 pump and a Gilson UV/VIS-155 detector (Gilson, Middleton, WI, USA).

### 2.2. Bacterial Isolation and Identification

The mealworm beetle used in this study was randomly selected as the specimens of insects raised at the Seoul Grand Park, Gwacheon-si, Gyeonggi Province, Republic of Korea. The selected specimen was identified as mealworm beetles, *Tenebrio molitor* Linnaues, based on external morphological characters. The mealworm beetle-associated actinomycete strain GG23 was isolated from the gut of adult mealworm beetles by using starch-casein agar (SCA), which contained 10 g of soluble starch, 0.3 g of casein, 2 g of KNO_3_, 0.05 g of MgSO_4_·7H_2_O, 2 g of K_2_HPO_4_, 2 g of NaCl, 0.02 g of CaCO_3_, 0.01 g of FeSO_4_·7H_2_O, and 18 g of agar in 1 L of distilled water. The strain was identified as *Streptomyces* sp. (GenBank accession number MT033037), which is closest to *Streptomyces cacaoi* (identity of 99.6%) (GenBank accession number NZ_MUBL01000000) based on 16S rRNA gene sequence analysis.

### 2.3. Cultivation and Extraction

The GG23 strain was initially cultured with 50 mL of modified PST medium (containing 5 g peptone, 5 g sucrose, and 5 g tryptone in 1 L distilled water) in a 125-mL Erlenmeyer flask. After 3 d of culture on a rotary shaker at 200 rpm and 30 °C, 5 mL of the culture was transferred to 200 mL of the same medium in a 500-mL Erlenmeyer flask. The culture was maintained for 3 d under the same conditions as the 50-mL stage, and 10 mL of the culture was inoculated into 1 L of PST medium in 2.8-L Fernbach flasks (60 each × 1 L, total volume 60 L) for 4-d incubation at 170 rpm and 30 °C. The whole culture was extracted twice with 120 L of ethyl acetate, and organic layer was separated and concentrated in vacuo to yield 25 g of dry material.

### 2.4. Isolation of Pentaminomycins C–E

The dried extract was re-suspended with celite in MeOH followed by drying in vacuo. The celite-adsorbed extract was loaded onto 2 g of a pre-packed C_18_ Sepak resin. The extract was fractionated using five different compositions of aqueous MeOH (i.e., 20, 40, 60, 80, and 100 vol% MeOH). Pentaminomycins C–E (**1**–**3**) eluted in the 80 and 100 vol% MeOH fractions. The fractions were dried, redissolved in MeOH, and filtered to remove insoluble particles. Pentaminomycins C–E were further purified by preparative reversed-phase HPLC with a Phenomenex Luna 10 μm C_18_(2) 250 × 21.20 mm column with a gradient system of 30–50 vol% aqueous MeCN over 30 min (flow rate: 10 mL/min, detection: UV 210 nm). Pentaminomycin D (**2**) eluted at 20 min under these HPLC conditions, whereas pentaminomycins C (**1**) and E (**3**) eluted together at 23 min. The eluates were subjected to semi-preparative reversed-phase HPLC using a YMC-Pack CN 250 × 10 mm, S-5 μm, 12 nm column with an isocratic system of 35 vol% MeCN containing 0.05% trifluoroacetic acid (flow rate: 2 mL/min, detection: UV 210 nm). In the final purification, pentaminomycins C (**1**) (5 mg), D (**2**) (3 mg), and E (**3**) (4 mg) were collected at retention times of 20, 28, and 33 min, respectively.

#### 2.4.1. Pentaminomycin C (**1**)

White powder; ${\left[\alpha \right]}_{D}^{20}$-50 (c 0.1, MeOH); UV (MeOH) λ_max_ (log ε) 218 (2.04), 281 (0.35) nm; IR (neat) ν_max_ 3302, 2965, 1673, 1537, 1444, 1199, 1140 cm^−1^; ^1^H and ^13^C NMR data, see Table 1; HR-FAB-MS *m/z* 718.4041 [M+H]^+^ (calcd. for C_37_H_52_N_9_O_6_ 718.4035).

#### 2.4.2. Pentaminomycin D (**2**)

White powder; ${\left[\alpha \right]}_{D}^{20}$-8 (c 0.1, MeOH); UV (MeOH) λ_max_ (log ε) 218 (2.20), 281 (0.36) nm; IR (neat) ν_max_ 3296, 2964, 1672, 1536, 1444, 1199, 1140 cm^−1^; ^1^H and ^13^C NMR data, see Table 1; HR-FAB-MS *m/z* 704.3876 [M+H]^+^ (calcd. for C_36_H_50_N_9_O_6_ 704.3879).

#### 2.4.3. Pentaminomycin E (**3**)

White powder; ${\left[\alpha \right]}_{D}^{20}$-31 (c 0.1, MeOH); UV (MeOH) λ_max_ (log ε) 216 (1.91), 282 (0.25) nm; IR (neat) ν_max_ 3300, 2964, 1672, 1536, 1445, 1199, 1140 cm^−1^; ^1^H and ^13^C NMR data, see Table 1; HR-FAB-MS *m/z* 752.3879 [M+H]^+^ (calcd. for C_40_H_50_N_9_O_6_ 752.3879).

### 2.5. Marfey’s Analysis of Pentaminomycins D and E (**2** and **3**)

A sample (1 mg) of pentaminomycin D (**2**) was hydrolyzed in 0.5 mL of 6 N HCl at 100 °C for 1 h. After hydrolysis, the reaction vial was cooled in an ice bucket for 3 min. After this time, the reaction solvent was evaporated in vacuo, and the hydrolysate containing the free amino acids was dissolved in 100 µL of 1N NaHCO_3_. Subsequently, 50 µL of a 10 mg/mL l-FDLA solution in acetone was added to the solution. The reaction mixture was stirred at 80 °C for 3 min, then 50 µL of 2N HCl was used to neutralize the reaction mixture, which was subsequently diluted using 300 µL of a 50 vol% aqueous MeCN solution. An aliquot (20 µL) of the reaction mixture was analyzed by LC/MS using a Phenomenex C_18_(2) column (Luna, 100 × 4.6 mm, 5 µm) under gradient solvent conditions (flow rate 0.7 mL/min; UV 340 nm detection; 10–60 vol% MeCN/H_2_O containing 0.1% formic acid over 50 min). LC/MS analysis indicated that during acid hydrolysis, *N*^5^-hydroxyarginine was converted to arginine. The l-FDLA derivatives of the two valine (37.5 and 44.6 min), tryptophan (41.0 min), arginine (22.6 min), and phenylalanine (47.5 min) residues of pentaminomycin D were detected by LC/MS analysis. The same procedure was performed for authentic l-and d-Val, Trp, Arg, and Phe to compare the retention times with those of the amino acids from **2** (Table S1 and Figure S17). The absolute configurations of pentaminomycin E (**3**) were also established in the same manner.

### 2.6. Genome Analysis and the Biosynthetic Pathway

Whole genome sequencing of the GG23 strain was performed using PacBio RS II (Chunlab, Inc., Seocho-gu, Seoul, Korea).The sequencing data were assembled with PacBio SMRT Analysis v. 2.3.0, using a hierarchical genome assembly process (HGAP) protocol. Nucleotide sequences of the *Streptomyces* sp. GG23 genomes were generated in three contigs with a total of 8,666,993 base pairs. Gene prediction was performed using Prodigal v. 2.6.2, and sequences were annotated with EggNOG v. 4.5, Swissprot, KEGG, and SEED (Chunlab, Inc., Seocho-gu, Seoul, Republic of Korea) The biosynthetic gene clusters (BGCs) were analyzed using antiSMASH v. 5.0 [19].

### 2.7. Autophagic Flux Assay

To examine cellular autophagic flux after treatment of pentaminomycins, HEK293 cells (≈80% confluence) were treated with 0, 3.125, 6.25, 12.5, or 25 µM pentaminomycin C, D, or E for 8 h. Whole cell extracts were prepared using RIPA buffer (50 mM Tris-HCl (pH 8.0), 1% of NP-40, 0.5% of deoxycholate, 0.1% of sodium dodecyl sulfate (SDS), and 150 mM NaCl) supplemented with protease inhibitor cocktails. The lysates were then centrifuged at 16,000× *g* for 30 min at 4 °C. The supernatants were separated by SDS-PAGE and transferred to a polyvinylidene difluoride (PVDF) membrane (Merck Millipore, Darmstadt, Germany). Subsequently, the membranes were blocked with 5% non-fat milk and probed with the following antibodies: anti-LC3 (L7543, MilliporeSigma, St. Louis, MO, USA), anti-SQSTM1 (sc-28359, Santa Cruz Biotechnology, Dallas, TX, USA), anti-GABARAPL1 (D5R9Y, Cell Signaling Technology, Dallas, TX, USA), and anti-GAPDH (A1978, MilliporeSigma). The membranes were then incubated with a horseradish peroxidase-conjugated anti-mouse IgG antibody (81-6720, Invitrogen, Carlsbad, CA, USA) or an anti-rabbit IgG antibody (G21234, Invitrogen), and visualized using an ECL system (Thermo Fisher Scientific, Waltham, MA, USA).

### 2.8. Cytotoxicity Assays

The cell viability was assessed using the CellTiter-Glo Luminescent Cell Viability Assay (Promega) kit as previously described [20]. More specifically, the cells were grown in a black wall/clear-bottom 96-well plate and treated with either pentaminomycins C–E (20 µM; **1**–**3**), menadione (25 µM), or combinations of pentaminomycins and menadione for 8 h at the indicated concentrations. After the addition of luminescence substrates in the same volume as the cell culture medium, the mixtures were incubated for 2 min at room temperature on a shaker, followed by 10 min incubation at room temperature to stabilize the luminescence signal prior to measurement.

## 3. Results and Discussion

### 3.1. Structural Elucidation

Pentaminomycin C (**1**) was purified as a white powder, and its molecular formula was established to be C_37_H_51_N_9_O_6_ based on HRFABMS data along with ^1^H and ^13^C NMR data (Table 1). Further analysis of the NMR spectra (Figures S1–S5) confirmed this compound as the previously reported cyclic peptide, pentaminomycin C [18], which consists of five amino acids: leucine, valine, tryptophan, *N*^5^-hydroxyarginine, and phenylalanine. The sequence of the amino acids was confirmed as leucine-valine-tryptophan-*N*^5^-hydroxyarginine-phenylalanine by HMBC correlations as reported in the literature [18].

Pentaminomycin D (**2**) was isolated as a white powder. Based on HRFABMS and NMR data, the molecular formula of 2 was determined to be C_36_H_49_N_9_O_6_ with 17 double bond equivalents. Based on this molecular formula, pentaminomycin D (**2**) possesses one less CH_2_ group than **1**. The ^1^H NMR spectrum of **2** (Figure S6) showed the presence of five exchangeable amide NH groups (δ_H_ 8.85, 8.63, 8.45, 7.50, and 7.23) and five α-protons (δ_H_ 4.52, 4.28, 4.16, 4.12, and 3.70) in the amino acid residues, suggesting that **2** was also a pentapeptide-derived compound. The ^13^C NMR spectrum (Figure S7) also confirmed **2** to be a peptidic metabolite through its five carbonyl signals (δ_C_ 171.7, 171.4, 171.4, 170.7, and 170.4) and five α-carbon signals (δ_C_ 60.2, 57.5, 55.3, 53.5, and 52.9), which is consistent with the ^1^H NMR spectrum. Further analysis of the ^13^C NMR spectrum identified 15 sp^2^ carbon atoms (δ_C_ 157.4–110.2) and 11 aliphatic carbon atoms (δ_C_ 50.5–18.3). The odd number of sp^2^ carbon atoms indicated the existence of an imine-type functional group. Assuming that pentaminomycin D (**2**) possessed an imine group, eight double bonds and five carbonyl groups accounted for 13 double bond equivalents out of 17, suggesting that this metabolite possessed four rings.

**Figure 2:**
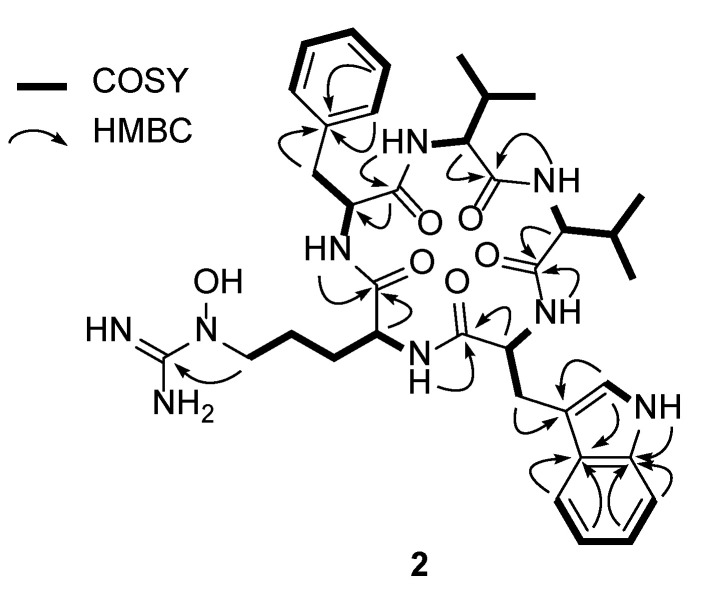
Key COSY and HMBC correlations of pentaminomycin D (**2**).

Analysis of the 1D (^1^H and ^13^C) and 2D (HSQC, COSY, and HMBC) NMR spectroscopic data (Figures S6–S10) of **2** identified the amino acid residues (Figure 2). More specifically, the conspicuous 1′-NH moiety (δ_H_ 10.78) was found to have a COSY correlation with H-2′ (δ_H_ 7.17), connecting C-1′ and C-2′. C-2′ was located adjacent to C-3′, as determined by the H-2′/C-3′ HMBC correlation. The ^3^*J*_HH_ correlations of H-4′ (δ_H_ 7.51), H-5′ (δ_H_ 6.98), H-6′ (δ_H_ 7.04), and H-7′ (δ_H_ 7.30), and their ^1^H-^1^H coupling constants (*J* = 7.5 Hz), allowed the construction of an *ortho*-substituted 6-membered aromatic ring. HMBC signals from 1′-NH and H-7′ to C-7′a (δ_C_ 136.1) and from H-2′ and H-5′ to C-3′a (δ_C_ 126.8) secured the 1′-NH-C-7a’ and C-3′-C-3′a connectivity and allowed the elucidation of the indole structure. H_2_-3 (δ_H_ 3.19 and 2.91) displayed HMBC correlations with C-3′ (δ_C_ 110.2), indicating C-3 methylene substitution at C-3′. In addition, 2-NH (δ_H_ 8.63)/H-2 (δ_H_ 4.28) and H-2/H_2_-3 COSY and H-2/C-1 HMBC correlations confirmed the presence of a tryptophan unit, while an array of COSY correlations starting from an NH group (δ_H_ 7.50) to H_3_-4 and H_3_-5 confirmed the existence of a valine residue. In a similar manner, COSY correlations from an amide NH moiety (δ_H_ 8.45) to a dimethyl group allowed the elucidation of an addition valine unit. Furthermore, correlation of the α-proton at δ_H_ 4.52 with an amide proton (δ_H_ 8.85) and β-protons (δ_H_ 2.98 and 2.79) was also observed, as were HMBC correlations between these β-protons with the quaternary C-1′ carbon atom of the aromatic ring (δ_C_ 138.0) and the C-2′ carbon atom (δ_C_ 129.0). Two overlapping methine carbon peaks (2 × CH) at δ_C_ 129.0 and 128.0, and the second-order signals observed for 5H indicated the presence of a phenylalanine residue. This unit was further assigned by HMBC correlations from H-2′ and H-5′ to C-6′ (δ_C_ 126.2) and from H-3′ and H-6′ to C-1′. The presence of the *N*^5^-hydroxyarginine moiety was deciphered by consecutive COSY correlations from NH (δ_H_ 7.23) to H-5 (δ_H_ 3.42). The H_2_-5 methylene protons showed COSY correlations only with H_2_-4 (δ_H_ 1.33 and 1.15), placing the methylene unit at the terminus of this spin system. Moreover, the ^13^C chemical shift of C-5 (δ_C_ 50.5) indicated that this carbon atom was bound to a nitrogen atom. This partial structure and the four elucidated amino acids (Trp, Phe, and two Val’s) accounted for the C_35_H_45_N_6_O_5_ portion of the molecular formula C_36_H_49_N_9_O_6_, thereby leaving a CH_4_N_3_O unit for structural elucidation. Thus, this last carbon (δ_C_ 157.4), which was preliminarily diagnosed as an imine carbon, was correlated with H_2_-5. Its chemical shift is typical for guanidine carbon, and the presence of three broad singlet protons at δ_H_ 7.50 confirmed the presence of a guanidine group containing the *N*^5^-OH group (δ_H_ 10.55), thereby indicating that this last fragment is an *N*^5^-hydroxyarginine residue.

The seven double bonds, one imine group, five carbonyl groups, and three ring structures of the Phe and Trp residues accounted for 16 double bond equivalents out of 17 for pentaminomycin D (**2**). An additional ring structure was therefore confirmed in the sequence analysis of the amino acids using the HMBC spectrum (Figure S10). More specifically, the HMBC correlations from the α-proton (δ_H_ 4.12) of Val-1 and the amide proton (δ_H_ 8.45) of Val-2 to the C-1 atom (δ_C_ 171.4) of Val-1 established the connectivity of Val-1 to Val-2. The connection of Val-2 to Trp was supported by the heteronuclear correlation from the NH moiety (δ_H_ 8.63) of Trp to the C-1 atom (δ_C_ 171.4) of Val-2 in the HMBC spectrum. An additional HMBC correlation from the amide NH group (δ_H_ 7.23) of *N*^5^-OH-Arg to the amide carbonyl carbon atom (δ_C_ 171.7) of Trp secured the sequence of Trp to *N*^5^-OH-Arg. Furthermore, the NH proton (δ_H_ 7.23) of *N*^5^-OH-Arg correlated with the amide carbonyl carbon (δ_C_ 170.7) of Phe in the HMBC spectrum, which established the linkage of arginine to phenylalanine. Lastly, the cyclized structure was completed by the confirmation of an HMBC correlation from the NH unit (δ_H_ 8.85) of Phe to the carbonyl carbon atom (δ_C_ 171.4) of Val-1, finally establishing the planar structure of **2** as a new cyclic pentapeptide.

Pentaminomycin E (**3**) was also purified as a white powder. The molecular formula of this compound was determined to be C_40_H_49_N_9_O_6_ based on HRFABMS and NMR data (Table 1). Comparing the NMR spectra of **3** with those of **1** and **2**, additional aromatic protons and carbons were observed, indicating the presence of an additional aromatic group in 3. Comprehensive analysis of the 1D and 2D NMR spectra (Figures S11–S15) confirmed the amino acid residues to be two phenylalanine residues, valine, tryptophan, and *N*^5^-hydroxyarginine. The amino acid sequence of the structure was subsequently determined by analysis of the HMBC correlations, and was confirmed to be Phe-1-Val-Trp-*N*^5^-OH-Arg-Phe-2. The new metabolites, namely pentaminomycins D and E (**2** and **3**), share cyclic pentapeptide features with pentaminomycins A and B, including Trp and *N*^5^-OH Arg [17]. However, pentaminomycins D and E incorporate Phe instead of Leu adjacent to *N*^5^-OH-Arg, unlike in the cases of pentaminomycins A and B [17]. In addition, pentaminomycin E was identified as the first congener bearing two Phe units in the pentaminomycin series.

To determine the absolute configurations of pentaminomycins D and E (**2** and **3**), acid hydrolysis and derivatization of the hydrolysates with Marfey’s reagent (*N*-(5-fluoro-2,4-dinitrophenyl)-l-leucine amide (l-FDLA)) were carried out [21]. By comparing the retention times from LC/MS analysis of the l-FDLA derivatives with the same reaction products of authentic l and d amino acids, the absolute configurations of the α-carbons were determined. The absolute configurations of the amino acids in pentaminomycin D (**2**) were established as l-valine, d-valine, l-tryptophan, *N*^5^-hydroxy-l-arginine, and d-phenylalanine. In a similar process, the absolute configurations of the amino acid residues present in pentaminomycin E (**3**) were determined to be d-valine, l-tryptophan, *N*^5^-hydroxy-l-arginine, l-phenylalanine, and d-phenylalanine. Since two valine residues of the opposite configuration exist in **2**, whereas **3** contains both l- and d-phenylalanine residues, the exact assignments of the configurations were subjected to genomic analysis of the biosynthetic gene cluster for the pentaminomycins because the l- and d-Val units in **2** and the l- and d-Phe residues in **3** are not distinguishable by NMR spectroscopic analysis.

### 3.2. Biosynthetic Pathway

**Figure 3:**
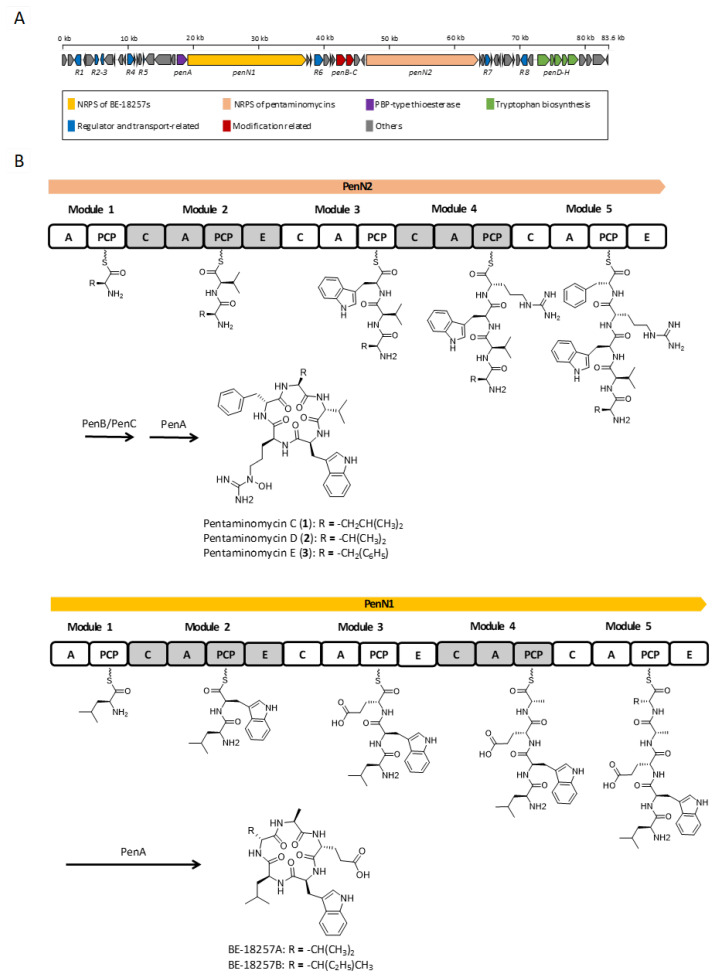
Proposed biosynthesis pathway for the BE-18257s and the pentaminomycins. (**A**) Genetic organization of putative biosynthetic gene cluster of the pentaminomycins. (**B**) Putative biosynthetic pathway for the pentaminomycins and the BE-18257s with the nonribosomal peptide synthetase (NRPS) modular organization. C, condensation domain; A, adenylation domain; PCP, peptidyl carrier protein; E, epimerase domain.

Analysis of the whole genome sequence of the *Streptomyces* sp. GG23 strain identified the putative biosynthetic gene cluster responsible for the pentaminomycins. The 8.7 Mb draft genome consisting of three contigs was analyzed using antiSMASH 5.0 [19]. In total, 31 gene clusters were involved in the biosynthesis of polyketides, nonribosomal peptides, and terpenes (Table S2). The BGC of the pentaminomycins showed a high similarity to a previous report into pentaminomycin C [18]. The total length of the BGC is approximately 83.6 kb encompassing 53 open reading frames (Table S3) including two NRPS genes, three post-modification genes, seven transport and regulatory genes, and five tryptophan biosynthesis genes (Figure 3A).

The NRPS (non-ribosomal peptide synthetase) gene for the pentaminomycins is *penN2*, which encodes five NRPS modules. Each module incorporates an amino acid to produce a pentapeptide chain. The first module without the epimerase domain flexibly recruits an amino acid of the group l-valine, l-leucine, and l-phenylalanine. The amino acid introduced by the second module is fixed as valine, whose absolute configuration is the d form because of the action of the epimerase domain in this module. This shows that the Val-2 residue introduced by module 2 has a d configuration and the other valine unit (Val-1) is in the l form in pentaminomycin D (**2**). Accordingly, l-Trp should be tethered after d-Val by module 3, and the arginine moiety is connected by module 4. The peptide chain is completed after the linkage of the last amino acid, d-Phe, by module 5 with the action of the epimerase domain. This also confirmed the absolute configuration of pentaminomycin E (**3**), in which Phe-2 is present in the d form, whereas Phe-1 possesses the l configuration. Post-modular modification by processes such as hydroxylation and cyclization finalized the biosynthesis of the pentaminomycins. *N*-hydroxylation on the arginine unit is possibly facilitated by the cytochrome P450 enzymes PenB and/or PenC. Cyclization of the pentapeptide chain was proposed to be catalyzed by serine hydrolase *penA* in the previously reported biosynthesis of pentaminomycin C [18]. However, our detailed analysis found that *penA* is the coding gene for penicillin binding protein (PBP)-type thioesterase (TE). PBP-type TE or the standalone cyclase is reported as a peptidyl cyclase included in the β-lactamase superfamily [22,23]. Cyclic peptides that use PBP-type TEs have been previously reported, including desotamide [24,25,26], surugamide [27], ulleungmycin [28], noursamycin [29], curacomycin [30], and mannopeptimycin [31]. These compounds share a structurally common feature in that the initial NRPS module must introduce an l-amino acid, while the terminal module recruits a d-amino acid. This is due to the fact that the structure of PBP-type TE is analogous to the penicillin-binding protein [32]. The penicillin-binding protein detects the d-alanyl-d-alanine moiety in peptidoglycan precursors to contribute to transpepdidation for cell wall construction in bacteria [33]. Similarly, PBP-type TE also detects the d-amino acid at the C-terminus of the NRPS peptide chain and catalyzes peptidyl macrocyclization. Based on the NRPS modules, the biosynthesis of the pentaminomycins starts with an l-amino acid (l-Val, l-Leu, or l-Phe) and ends with d-Phe, facilitating the cyclization by PBP-type TE (PenA) (Figure 3B).

The BGC contains another NRPS gene, namely *penN1*, which is located close to the *penN2* gene (Figure 3B). Detailed analysis of the sequence revealed that *penN1* is also composed of five NRPS modules biosynthesizing another series of cyclic peptides, i.e., BE-18257A and B, which were reported to be endothelin-binding inhibitors [34]. In our chemical analysis of *Streptomyces* sp. GG23 based on LC/MS data, the production of these cyclic peptides was detected (Figure S16). The pentapeptide chains of BE-18257s are assumed to be cyclized by PenA because *penN1* does not possess canonical TE at the end of the NRPS module and no other PBP-type TE genes rather than *penA* were identified in the BGC. Furthermore, the NRPS gene of the BE-18267s initially introduces l-Leu and completes the biosynthesis of the pentapeptide chain with d-Val or d-Leu, which is suitable for the utilization of PBP-type TE. Additionally, tryptophan biosynthetic genes (i.e., *penD–H*) exist in the BGC [35]. The pentaminomycins and BE-18257s both contain tryptophan in their structures, and so it is hypothesized that the Trp units of these different cyclic peptides share the Trp biosynthetic gene. Even though sharing of the PBP-type TE and Trp biosynthetic genes has to be proven by a follow-up study, if confirmed, the above example could be considered an unusual case in which independent NRPSs share core genes for their biosynthesis.

Based on the determined structures for pentaminomycins C–E (**1**–**3**), the amino acid introduced by module 1 appears to be flexible. The Leu, Val, or Phe variation could be explained by the amino acid sequence of the binding pocket providing substrate specificity to the adenylation domain [36]. Eight amino acid residues exist in the binding pocket to determine the specificity, whereby the amino acid residues of the binding pocket of module 1 are Asp235-Ala236-Leu239-Trp278-Met299-Gly301-Val322-Val330. Although many variables exist in the binding pockets, one of representative sequence for aromatic amino acids such as phenylalanine and tryptophan is Asp235-Ala236-Leu239-Val278-Met299-Gly301-Ala322-Val330. Comparing the two residue sequences, the only differences were that the Val278 and Ala322 residues in the typical binding pocket were replaced with Trp278 and Val322, respectively, in module 1 of *penN2*. This means that the adenylation domain of module 1 acts to incorporate an aromatic amino acid, such as Phe or Trp. However, because of these substitutions (Val278→Trp and Ala322→Val), module 1 potentially gains promiscuity to recruit variable hydrophobic amino acids, such as Leu, Val, or Phe, as a substrate. These amino acid sequence changes result in a smaller pocket volume, thereby avoiding larger aromatic side chains, such as that of Trp, which is consistent with the determined structures of pentaminomycins C–E. Indeed, instead of Trp, the smaller pocket seems to prefer smaller hydrophobic amino acids, such as Val or Leu, thereby accounting for the production of pentaminomycins C and D.

### 3.3. Evaluation of the Bioactivity

**Figure 4:**
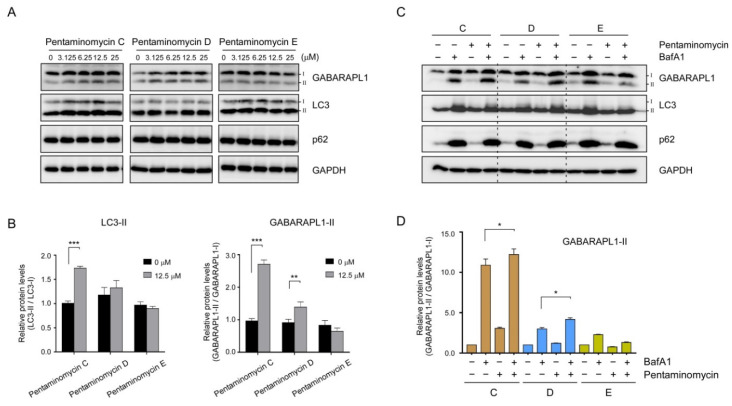
Effects of pentaminomycins C–E (**1**–**3**) on cellular autophagy in mammalian cells. HEK293T cells were treated with various concentrations of pentaminomycins for 8 h. (**A**) Whole cell lysates were harvested and subjected to SDS-PAGE followed by immunoblotting against the indicated antibodies. (**B**) Quantification of LC3-II and GABARAPL1-II in the presence of pentaminomycins (12.5 µM) using the multiple immunoblot images. Data were normalized to those of non-lipidated proteins. Data represent mean ± SD from three independent experiments. **, *p* < 0.01 and ***, *p* < 0.001 (one-way analysis of variance (ANOVA) with Bonferroni’s multiple comparison test). (**C**) Pentaminomycins C and D, but not E, induce global cellular autophagy. HEK293T cells were cotreated with pentaminomycins C–E (20 μM) and a downstream autophagy inhibitor bafilomycin A1 (BafA1; 100 nM) for 12 h. (**D**) Quantification of GABARAPL1-II normalized to GABARAPL1-I in the presence or absence of pentaminomycins and BafA1. *, *p* < 0.05 (one-way ANOVA with Bonferroni’s multiple comparison test).

The majority of amino acids constituting the pentaminomycins are non-polar residues that are expected to penetrate mammalian cells by simple diffusion and affect the membrane dynamics in the cell. In addition, similar cyclic peptides with lipophilic side chains often induce autophagy in cultured human cells [37,38]. We proceeded to examine whether pentaminomycins C–E affected the cellular autophagic flux by monitoring the conjugation of phosphatidylethanolamine (PE) to ATG8 proteins, such as microtubule-associated protein light chain 3 (LC3) and γ-aminobutyric acid receptor-associated protein (GABARAP), which is the hallmark of autophagy induction [39]. When HEK293T cells were treated with pentaminomycins C and D (**1** and **2**), the levels of lipidated forms of LC3 and GABARAPL1 (LC3-II and GABARAPL1-II, respectively) were significantly elevated in a moderate dose-dependent manner, while pentaminomycin E (**3**) did not exert a similar phenomenon (Figure 4A,B). The key autophagic receptor p62/SQSTSM1 remained virtually unchanged after compound treatment (Figure 4A).

Changes in the autophagic flux manifested as elevated levels of cellular LC3-II and GABARAPL1-II after treatment with pentaminomycins C and D may originate from either an increased overall cellular autophagy or the reduced autolysosomal degradation of LC3-II and GABARAPL1-II. To determine the molecular mechanism, the cells were treated with BafA1, which inhibits autophagy at a late stage by blocking the fusion between the autophagosome and the lysosome, prior to treatment with the pentaminomycins. We observed a modest increase in GABARAPL1-II upon exposure to pentaminomycins C (**1**) and D (**2**), but not pentaminomycin E (**3**), when the cells were cotreated with BafA1 (Figure 4C,D). Taken together, our data largely point that pentaminomycins C and D induce global autophagy instead of inhibiting cellular autophagic flux, although the underlying molecular mechanism and direct target molecules of pentaminomycins should therefore be determined.

**Figure 5:**
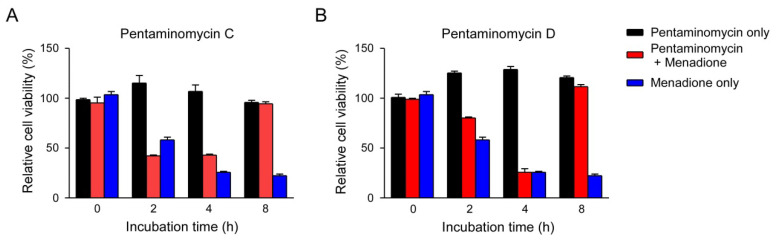
Alleviation of menadione-mediated cytotoxicity by (**A**) pentaminomycin C and (**B**) pentaminomycin D. Oxidative stress was induced by menadione (25 μM) for the indicated time periods in HEK93 cells, which were cotreated with either pentaminomycin C or D. The relative cell viability was assessed using the CellTiter-Glo assay and the values are represented as mean ± SD (*n* = 3).

Due to the fact that autophagy contributes to the degradation of oxidized proteins [40], we examined the effect of pentaminomycins C and D (**1** and **2**) on the oxidative stress induced by menadione [41]. Measurement of the cell viability based on the intracellular ATP levels revealed that the autophagy inducers, pentaminomycins C and D, potently protected HEK293 cells against menadione-induced cytotoxicity (Figure 5). Pentaminomycins C and D showed significantly reduced cell death after 4 and 2 h cotreatment with menadione, respectively. These protective effects were more prominent with longer pentaminomycin incubation times (Figure 5), thereby suggesting that autophagy induction by the pentaminomycins may accelerate oxidized protein clearance in cells and be beneficial in terms of cell protection under oxidative stress. However, it has yet to be determined whether natural compounds originating from mealworm beetle-associated bacteria can delay the pathologic process involving oxidatively damaged proteins, such as neurodegeneration and aging. Our results may therefore offer a novel strategy to modulate cellular autophagy and oxidative stress responses in cells.

## 4. Conclusions

Alteration of the culture conditions by changing the composition of culture medium, for a *Streptomyces* strain isolated from the gut of the mealworm beetle, *Tenebrio molitor*, enabled the production of cyclic pentapeptides, pentaminomycins C–E (**1**–**3**). The structures of **1**–**3** were assigned by combinational spectroscopic analysis. In addition, Marfey’s analysis and bioinformatic investigations of the nonribosomal peptide synthetase (NRPS) biosynthetic gene cluster established the absolute configurations of the new metabolites, pentaminomycins D and E. Detailed sequence analysis of the adenylation domains in the NRPS modules revealed that the structural variations among **1**–**3** originate from the low specificity for hydrophobic amino acids in module 1. In addition, it was found that cyclization of the pentaminomycins can be catalyzed by a penicillin binding protein (PBP)-type thioesterase (TE), which is a noncanonical TE requiring l- and d-amino acids in the starting and terminal units, respectively. Pentaminomycins C and D (**1** and **2**), but not pentaminomycin E (**3**), exhibited significant autophagy-inducing activity based on LC3 and GABARAPL1 lipidation in both the presence and absence of BafA1. Importantly, cells treated with pentaminomycins C and D showed enhanced resistance to the oxidative stress induced by menadione, providing strong evidence that activation of cellular autophagic flux antagonizes the harmful effects of oxidized proteins. Although the underlying molecular mechanism requires further elucidation, our findings collectively suggest that some pentaminomycins may exhibit therapeutic potential against diseases associated with chronic oxidative stress and incompetent cellular responses. The discovery of pentaminomycins C–E therefore indicates that biotechnical investigation into relatively unexploited insect-associated bacteria may be a promising strategy to explore microbial metabolites with unique biosynthetic pathways and interesting biological activities.

## Figures and Tables

**Table 1:** NMR data for **1**–**3** in DMSO-*d*_6_.

Pentaminomycin C (1)				Pentaminomycin D (2)				Pentaminomycin E (3)			
Position		δ_C_, type	δ_H_, mult (*J* in Hz)	Position		δ_C_, type	δ_H_, mult (*J* in Hz)	Position		δ_C_, type	δ_H_, mult (*J* in Hz)
l-Leu	1	172.2, C		l-Val	1	171.4, C		l-Phe	1	170.6, C	
	2	50.5, CH	4.40, ddd (15.0,9.0,7.0)		2	57.5, CH	4.12, dd (7.5,7.5)		2	52.8, CH	4.67, dt (9.0,7.5)
	3	41.1, CH_2_	1.34, m		3	30.7, CH	1.77, m		3	38.1, CH_2_	2.84, dd (13.5,7.0) 2.78, dd (13.5,7.5)
	4	24.2, CH	1.43, m		4	19.0, CH_3_	0.83, d (7.0)		1′	137.1, C	
	5	22.7, CH_3_	0.85, d (6.5)		5	18.3, CH_3_	0.76, d (7.0)		2′	129.1, CH	7.18, m
	6	22.0, CH_3_	0.82, d (6.5)		NH		7.50, m		3′	128.0, CH	7.22, m
	NH		7.55, d (9.0)						4′	126.2, C	7.17, m
									NH		7.69, d (9.0)
d-Val	1	171.3, C		d-Val	1	171.4, C		d-Val	1	171.2, C	
	2	59.9, CH	3.70, dd (10.0,7.5)		2	60.2, CH	3.70, dd (10.0,7.5)		2	59.9, CH	3.70, dd (10.0,7.5)
	3	28.5, CH	1.65, m		3	28.1, CH	1.64, m		3	28.5, CH	1.65, m
	4	19.0, CH_3_	0.75, d (6.5)		4	19.2, CH_3_	0.77, d (7.0)		4	19.0, CH_3_	0.75, d (6.5)
	5	18.5, CH_3_	0.34, d (6.5)		5	18.5, CH_3_	0.31, d (7.0)		5	18.5, CH_3_	0.34, d (6.5)
	NH		8.41, d (7.5)		NH		8.45, d (7.5)		NH		8.41, d (7.5)
l-Trp	1	171.7, C		l-Trp	1	171.7, C		l-Trp	1	171.6, C	
	2	55.3, CH	4.29, ddd (11.0,8.0,3.5)		2	55.3, CH	4.28, ddd (11.5,8.0,3.0)		2	55.3, CH	4.27, ddd (11.5,8.0,3.5)
	3	26.9, CH_2_	3.18, dd (14.5,3.0) 2.80, dd (14.5,12.0)		3	26.9, CH_2_	3.19, dd (14.5,3.0) 2.91, dd (14.5,11.5)		3	26.9, CH_2_	3.17, dd (14.5,3.0) 2.90, dd (14.5,11.5)
	2-NH		8.59, d (8.0)		2-NH		8.63, d (8.0)		2-NH		8.58, d (8.0)
	1′(NH)		10.78, br s		1′(NH)		10.78, br s		1′(NH)		10.76, br s
	2′	123.9, CH	7.17, m		2′	123.9, CH	7.17, m		2′	123.8, CH	7.16, m
	3′	110.2, C			3′	110.2, C			3′	110.2, C	
	3′a	126.8, C			3′a	126.8, C			3′a	126.8, C	
	4′	117.9, CH	7.51, d (8.0)		4′	117.8, CH	7.51, m		4′	117.8, CH	7.50, d (8.0)
	5′	118.3, CH	6.98, t (7.5)		5′	118.2, CH	6.98, dd (7.5,7.5)		5′	118.2, CH	6.97, dd (7.5,7.5)
	6′	120.8, CH	7.05, t (7.5)		6′	120.8, CH	7.04, dd (7.5,7.5)		6′	120.8, CH	7.04, dd (7.5,7.5)
	7′	111.3, CH	7.31, d (8.0)		7′	111.3, CH	7.30, d (8.0)		7′	111.3, CH	7.30, d (8.0)
	7′a	136.1, C			7′a	136.1, C			7′a	136.1, C	
*N*^5^-OH- l-Arg	1	170.4, C		*N*^5^-OH- l-Arg	1	170.4, C		*N*^5^-OH- l-Arg	1	170.4, C	
	2	52.8, CH	4.16, dt (7.0,7.0)		2	52.9, CH	4.16, dt (8.0,7.0)		2	53.0, CH	4.16, dt (7.0,7.0)
	3	28.1, CH_2_	1.53, m		3	28.2, CH_2_	1.53, m		3	28.2, CH_2_	1.52, m
	4	22.1, CH_2_	1.33, m, 1.18, m		4	22.0, CH_2_	1.33, m, 1.15, m		4	22.1, CH_2_	1.34, m, 1.17, m
	5	50.4, CH_2_	3.43, m		5	50.5, CH_2_	3.42, m		5	50.5, CH_2_	3.42, m
	*N^5^*-OH		10.49, br s		*N^5^*-OH		10.55, br s		*N^5^*-OH		10.47, s
	6	157.3, C			6	157.4, C			6	157.3, C	
	6-NH(3H)		7.45, br s		6-NH(3H)		7.50, br s		6-NH(3H)		7.43, br s
	NH		7.29, d (7.5)		NH		7.23, m		NH		7.26, d (7.5)
d-Phe	1	170.6, C		d-Phe	1	170.7, C		d-Phe	1	170.6, C	
	2	53.7, CH	4.46, ddd (9.0,9.0,6.0)		2	53.5, CH	4.52, ddd (9.5,8.5,6.0)		2	53.5, CH	4.47, ddd (9.0,9.0,5.5)
	3	34.2, CH_2_	2.96, dd (14.0,5.5) 2.80, dd (14.0,9.5)		3	33.9, CH_2_	2.98, dd (14.0,5.0) 2.79, dd (14.0,10.0)		3	34.0, CH_2_	2.93, dd (14.0,5.5) 2.76, dd (14.0,9.5)
	1′	137.9, C			1′	138.0, C			1′	137.9, C	
	2′, 6′	129.0, CH	7.24, m		2′, 6′	129.0, CH	7.24, m		2′, 6′	128.9, CH	7.20, m
	3′, 5′	128.0, CH	7.23, m		3′, 5′	128.0, CH	7.23, m		3′, 5′	128.0, CH	7.22, m
	4′	126.2, CH	7.17, m		4′	126.2, CH	7.17, m		4′	126.2, CH	7.17, m
	NH		8.85, d (8.0)		NH		8.85, d (8.0)		NH		8.89, d (8.5)

^1^H and ^13^C NMR data were recorded at 800 and 200 MHz, respectively (*J*: ^1^H-^1^H coupling constant).

## Supplementary Materials

The following are available online at https://www.mdpi.com/2076-2607/8/9/1390/s1, Figures S1–S5: ^1^H, ^13^C, COSY, HSQC, and HMBC NMR data of **1** in DMSO-*d*_6_, Figures S6–S10: ^1^H, ^13^C, COSY, HSQC, and HMBC NMR data of **2** in DMSO-*d*_6_, Figures S11–S15: ^1^H, ^13^C, COSY, HSQC, and HMBC NMR data of **3** in DMSO-*d*_6_, Table S1: LC/MS analysis of l-FDLA derivatives of **2** and **3**, Figure S16: Production of BE-18257s detected by LC/MS analysis. Figure S17: LC/MS chromatograms of l-FDLA derivatives of **2** and **3**, Table S2: antiSMASH output table of the whole genome analysis of *Streptomyces* sp. GG23, Table S3: Putative functions of ORFs in the pentaminomycin biosynthetic gene cluster.
