# Supplementary material for: Pentaminomycins C–E: Cyclic Pentapeptides as Autophagy Inducers from a Mealworm Beetle Gut Bacterium
Source: Microorganisms. 2020 Sep 10;8(9):1390. doi: 10.3390/microorganisms8091390 (PMC7565604; doi:10.3390/microorganisms8091390)
Supplement: Supplementary file 1 [file microorganisms-08-01390-s001.pdf]

Supplementary Material:

Pentaminomycins C–E: Cyclic Pentapeptides as Autophagy Inducers From a Mealworm Beetle Gut Bacterium

Sunghoon Hwang [1], Ly Thi Huong Luu Le [2], Shin-Il Jo [3], Jongheon Shin [1], Min Jae Lee [2,*] and Dong-Chan Oh [1,*]

[1] Natural Products Research Institute, College of Pharmacy, Seoul National University, 1 Gwanak-ro, Gwanak-gu, Seoul 08826, Republic of Korea; sunghooi@snu.ac.kr (S.H.); shinj@snu.ac.kr (J.S.); dongchanoh@snu.ac.kr (D.C.O.)

[2] Department of Biochemistry and Molecular Biology, Seoul National University College of Medicine, Seoul 03080, Republic of Korea; huongluuly94@snu.ac.kr (L.T.H.L.L.); minjlee@snu.ac.kr (M.J.L.)

[3] Welfare Division, Seoul Zoo, Seoul Grand Park, Gwacheon, Gyeonggi 13829, Republic of Korea; metather@seoul.go.kr (S.I.J.)

* Correspondence: minjlee@snu.ac.kr; Tel: +82-2-740-8254; Fax: +82-2-744-4534; dongchanoh@snu.ac.kr; Tel.: +82-2-880-2491; Fax: +82-2-762-8322.

Table of Contents

Figure S1. ^{1}H NMR spectrum (800 MHz) of pentaminomycin C (**1**) in DMSO-d_6.
Figure S2. ^{13}C NMR spectrum (200 MHz) of pentaminomycin C (**1**) in DMSO-d_6.
Figure S3. COSY spectrum (800 MHz) of pentaminomycin C (**1**) in DMSO-d_6.
Figure S4. HSQC spectrum (800 MHz) of pentaminomycin C (**1**) in DMSO-d_6.
Figure S5. HMBC spectrum (800 MHz) of pentaminomycin C (**1**) in DMSO-d_6.
Figure S6. ^{1}H NMR spectrum (800 MHz) of pentaminomycin D (**2**) in DMSO-d_6.
Figure S7. ^{13}C NMR spectrum (200 MHz) of pentaminomycin D (**2**) in DMSO-d_6.
Figure S8. COSY spectrum (800 MHz) of pentaminomycin D (**2**) in DMSO-d_6.
Figure S9. HSQC spectrum (800 MHz) of pentaminomycin D (**2**) in DMSO-d_6.
Figure S10. HMBC spectrum (800 MHz) of pentaminomycin D (**2**) in DMSO-d_6.
Figure S11. ^{1}H NMR spectrum (800 MHz) of pentaminomycin E (**3**) in DMSO-d_6.
Figure S12. ^{13}C NMR spectrum (200 MHz) of pentaminomycin E (**3**) in DMSO-d_6.
Figure S13. COSY spectrum (800 MHz) of pentaminomycin E (**3**) in DMSO-d_6.
Figure S14. HSQC spectrum (800 MHz) of pentaminomycin E (**3**) in DMSO-d_6.
Figure S15. HMBC spectrum (800 MHz) of pentaminomycin E (**3**) in DMSO-d_6.
Figure S16. Production of BE-18257A and B detected by LC/MS analysis.
Table S1. LC/MS analysis of L-FDLA derivatives of pentaminomycins D and E (**2**-**3**).
Figure S17. LC/MS chromatograms of Marfey's products of **2** and **3**.
Table S2. AntiSMASH output table of *Streptomyces* sp. GG23.
Table S3. Putative functions of ORFs in the pentaminomycin biosynthetic gene cluster.

Figure S1. ^{1}H NMR spectrum (800 MHz) of pentaminomycin C (**1**) in DMSO-d_6.

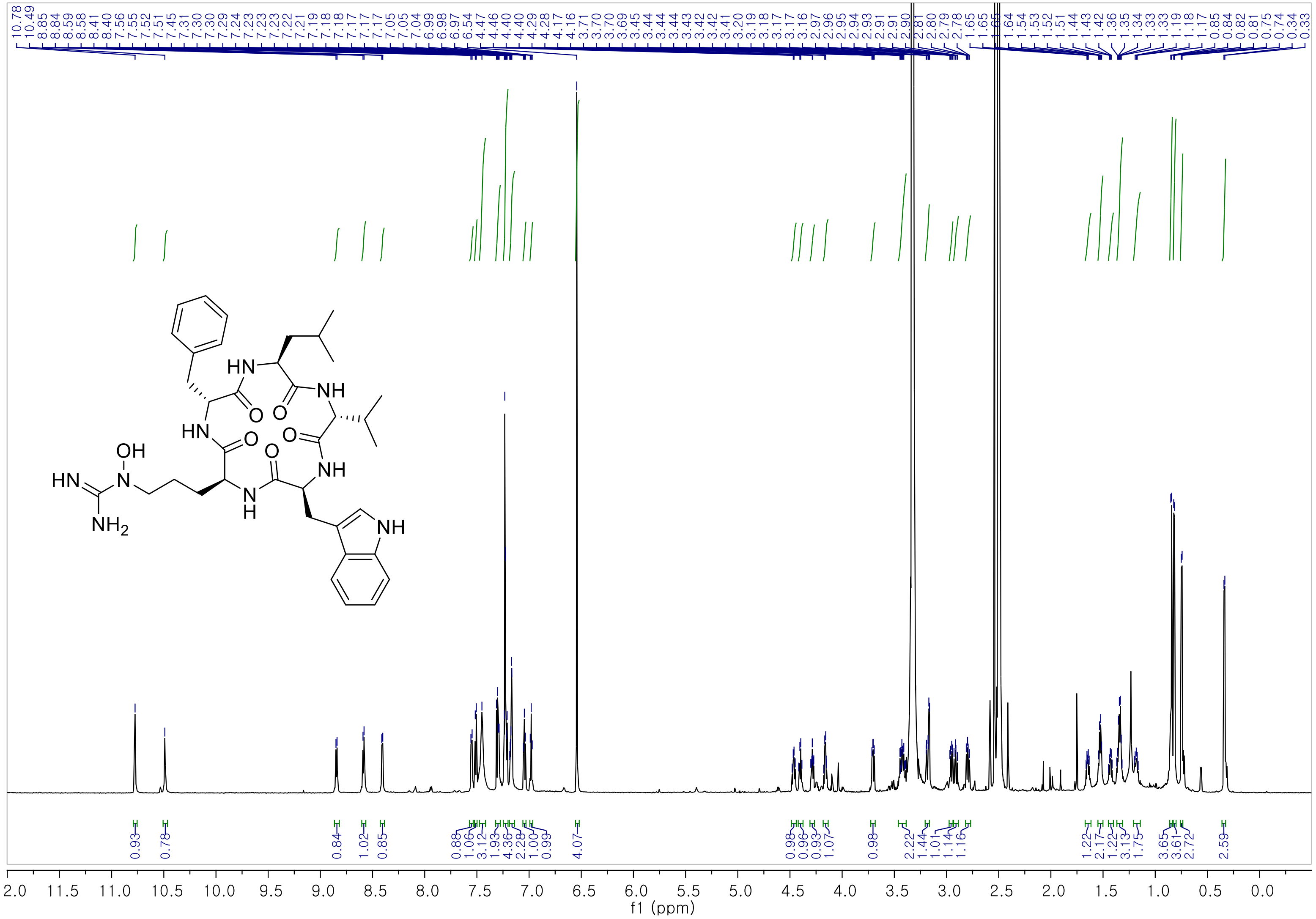

Figure S2. ^{13}C NMR spectrum (200 MHz) of pentaminomycin C (**1**) in DMSO-d_6.

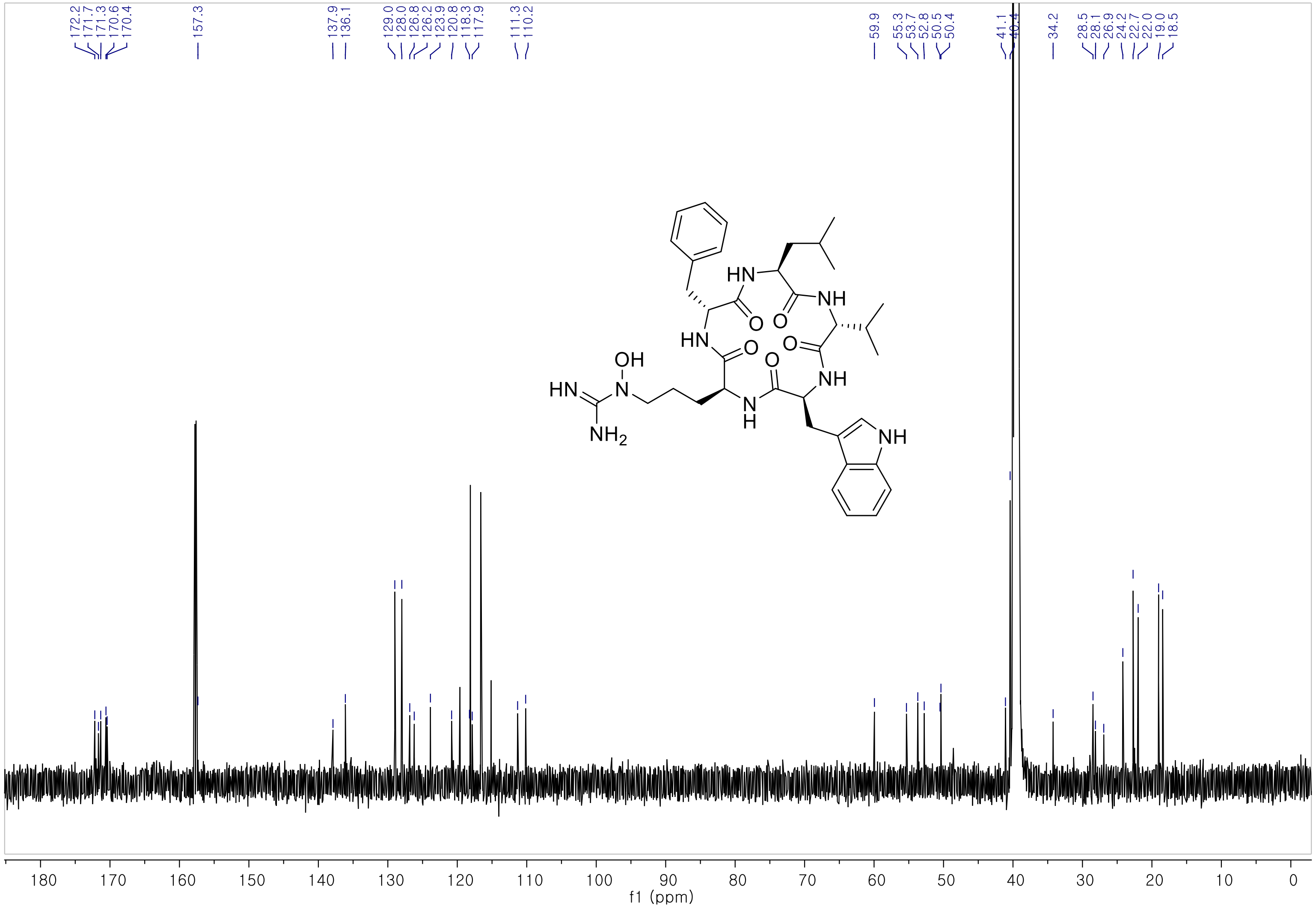

Figure S3. COSY NMR spectrum (800 MHz) of pentaminomycin C (**1**) in DMSO-d_6.

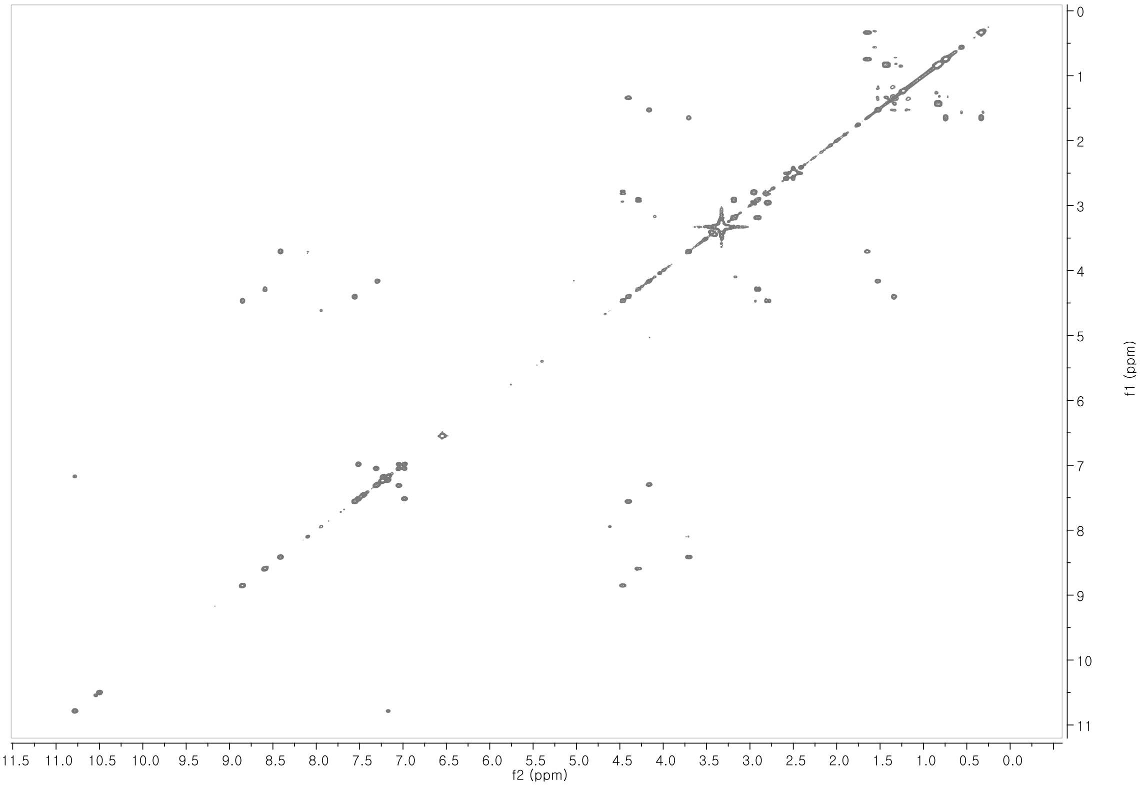

Figure S4. HSQC NMR spectrum (800 MHz) of pentaminomycin C (**1**) in DMSO-d_6.

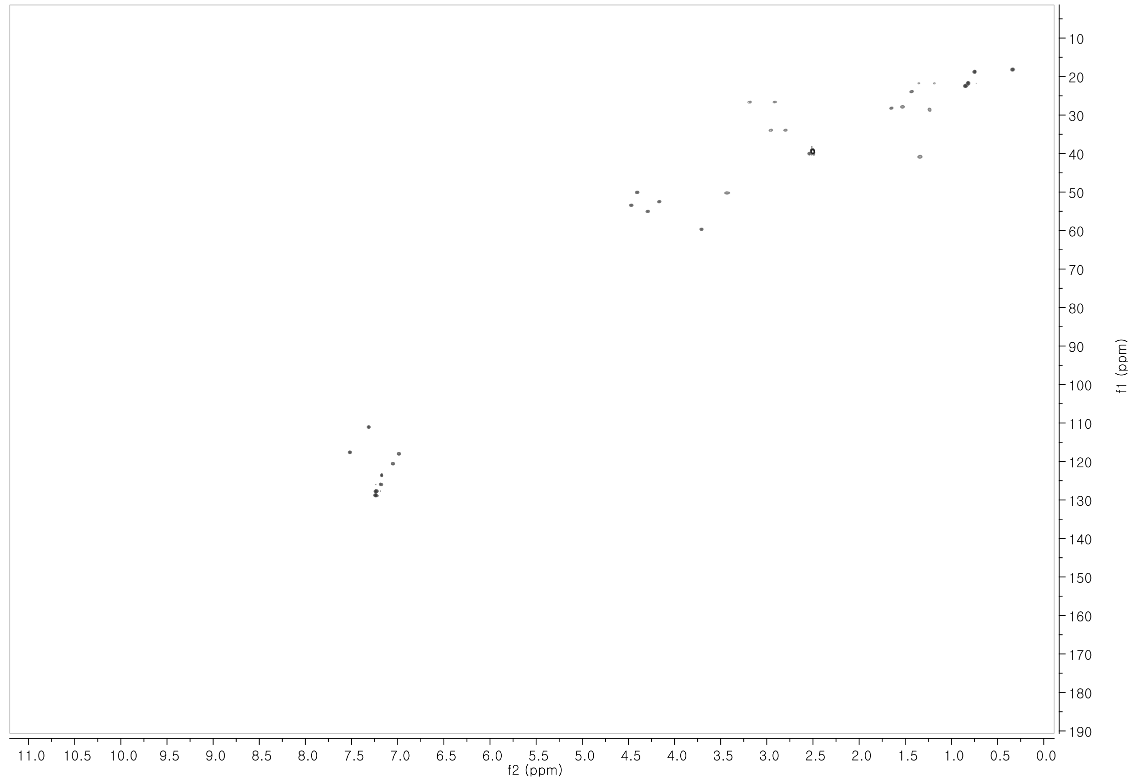

Figure S5. HMBC NMR spectrum (800 MHz) of pentaminomycin C (**1**) in DMSO-d_6.

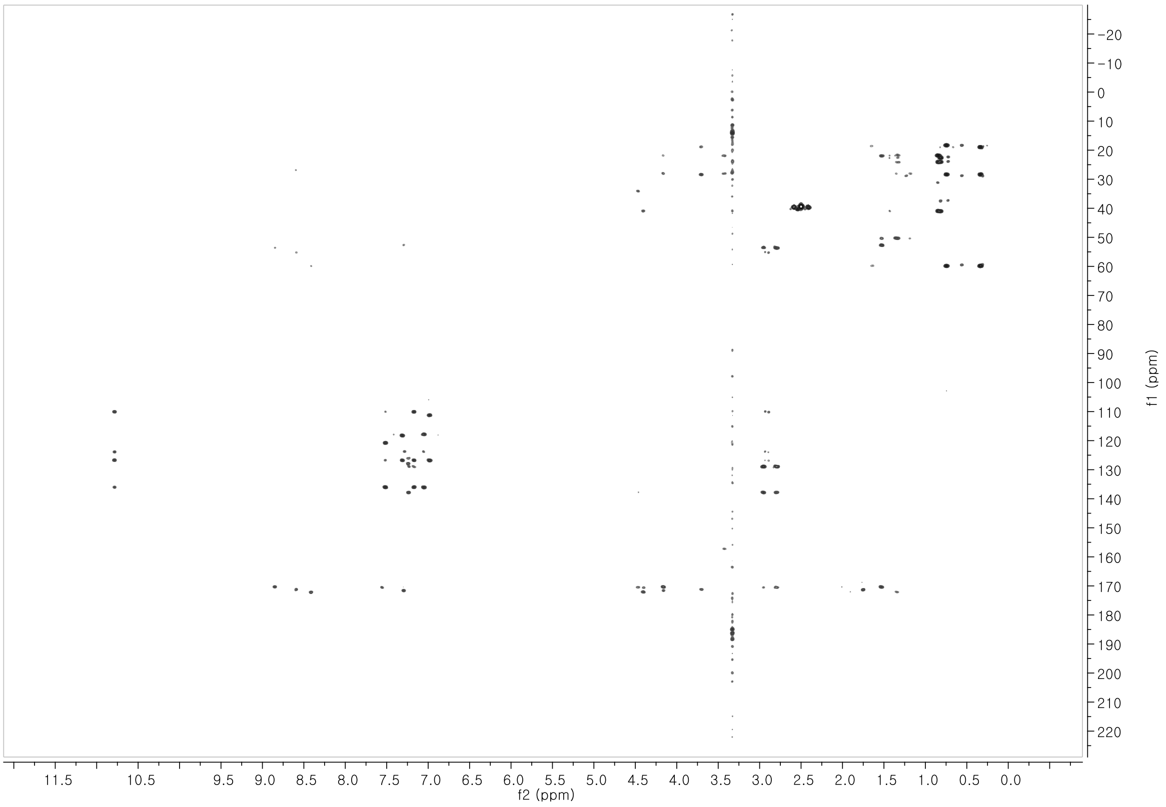

Figure S6. ^{1}H NMR spectrum (800 MHz) of pentaminomycin D (**2**) in DMSO-*d*6.

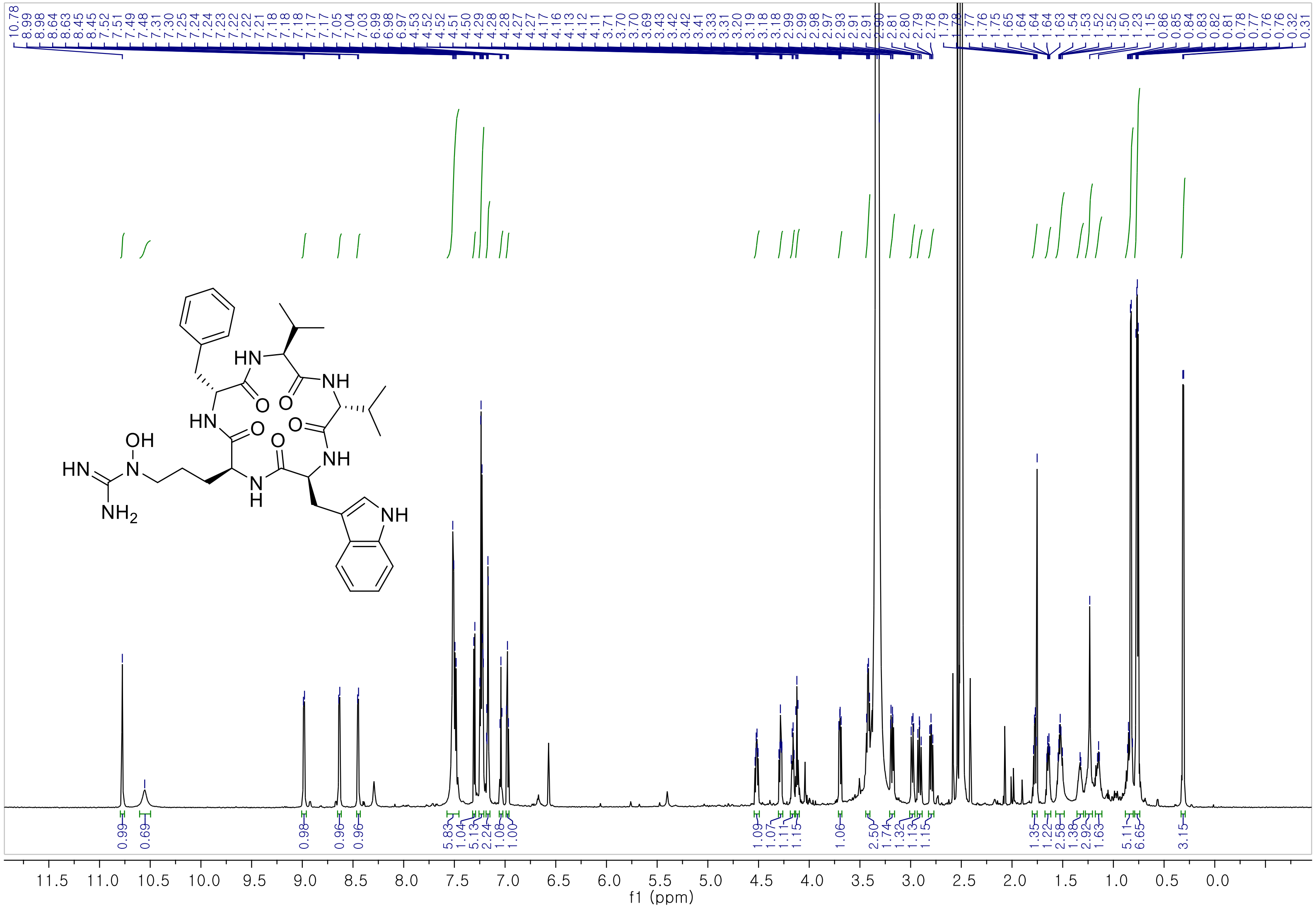

Figure S7. ^{13}C NMR spectrum (200 MHz) of pentaminomycin D (**2**) in DMSO-d_6.

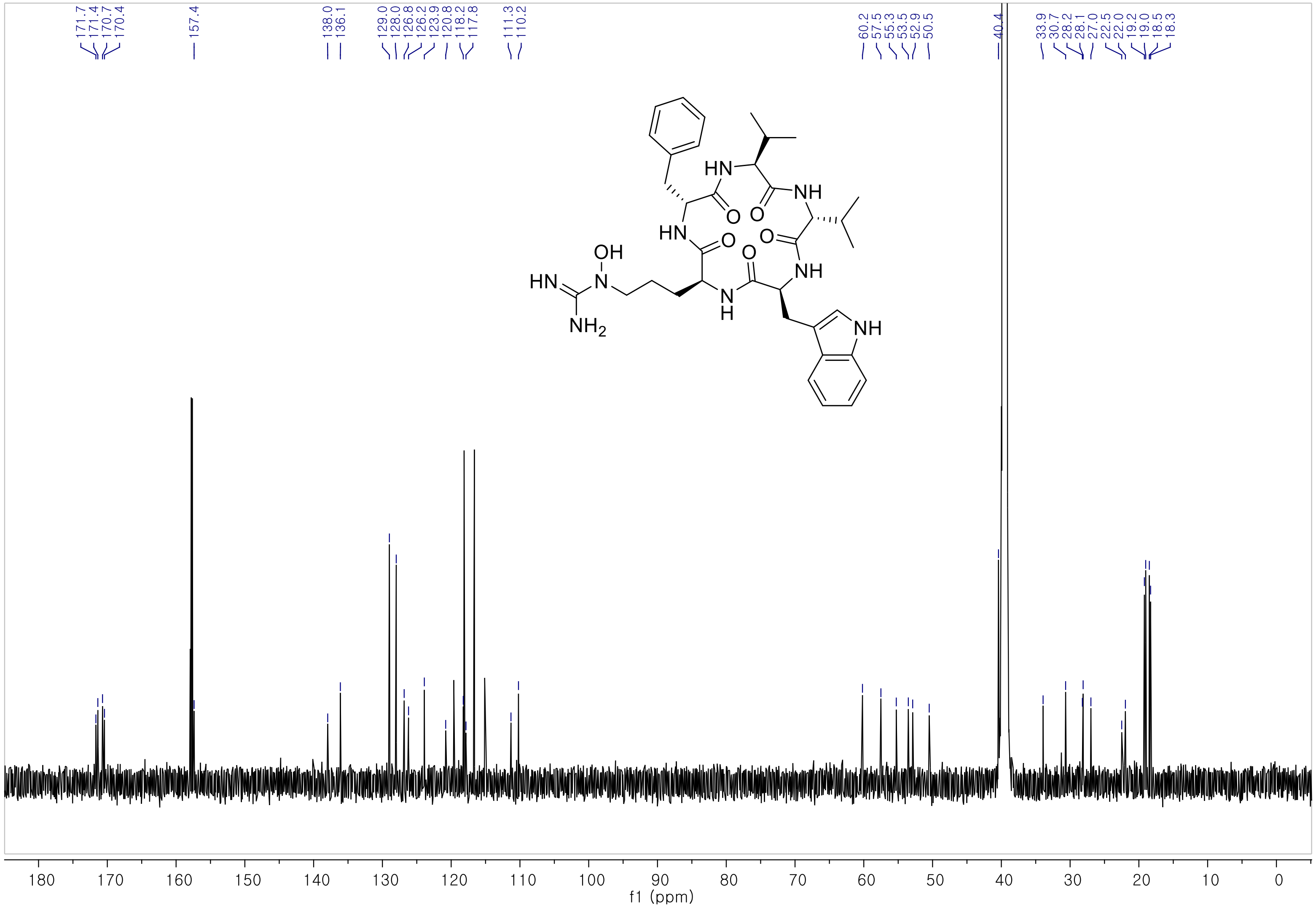

Figure S8. COSY NMR spectrum (800 MHz) of pentaminomycin D (**2**) in DMSO-d_6.

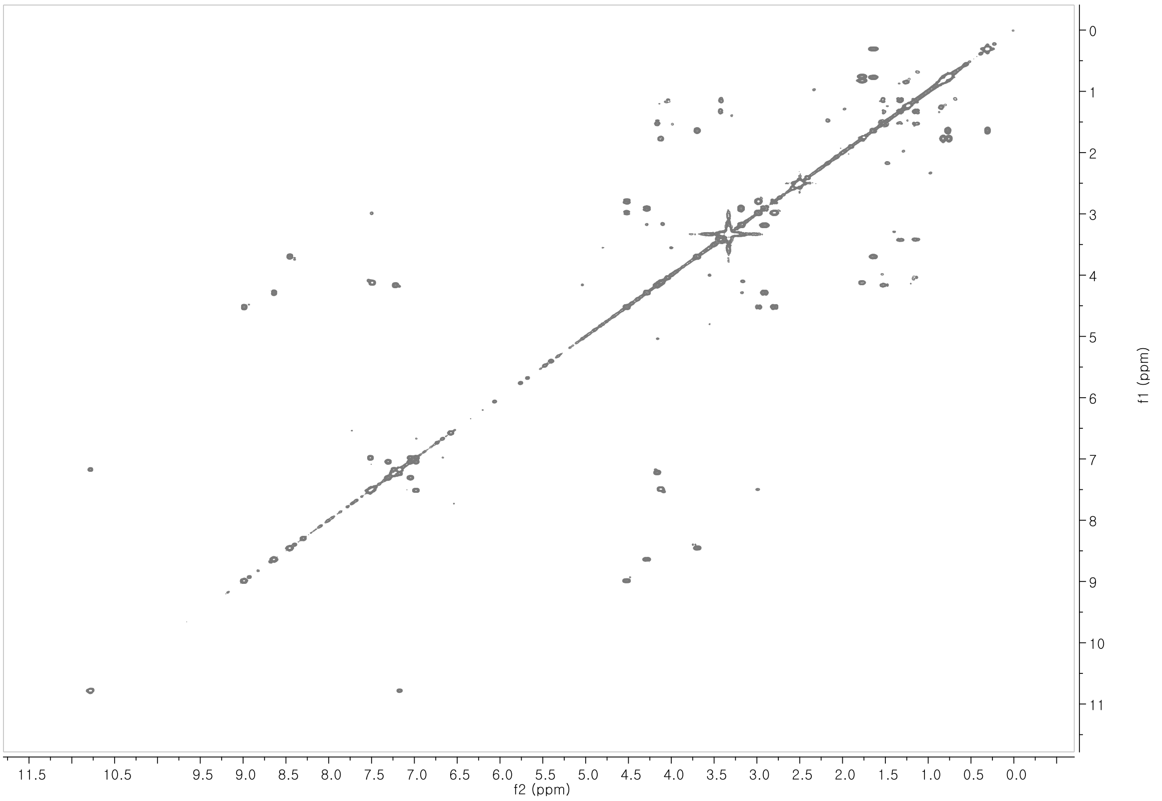

Figure S9. HSQC NMR spectrum (800 MHz) of pentaminomycin D (**2**) in DMSO-d_6.

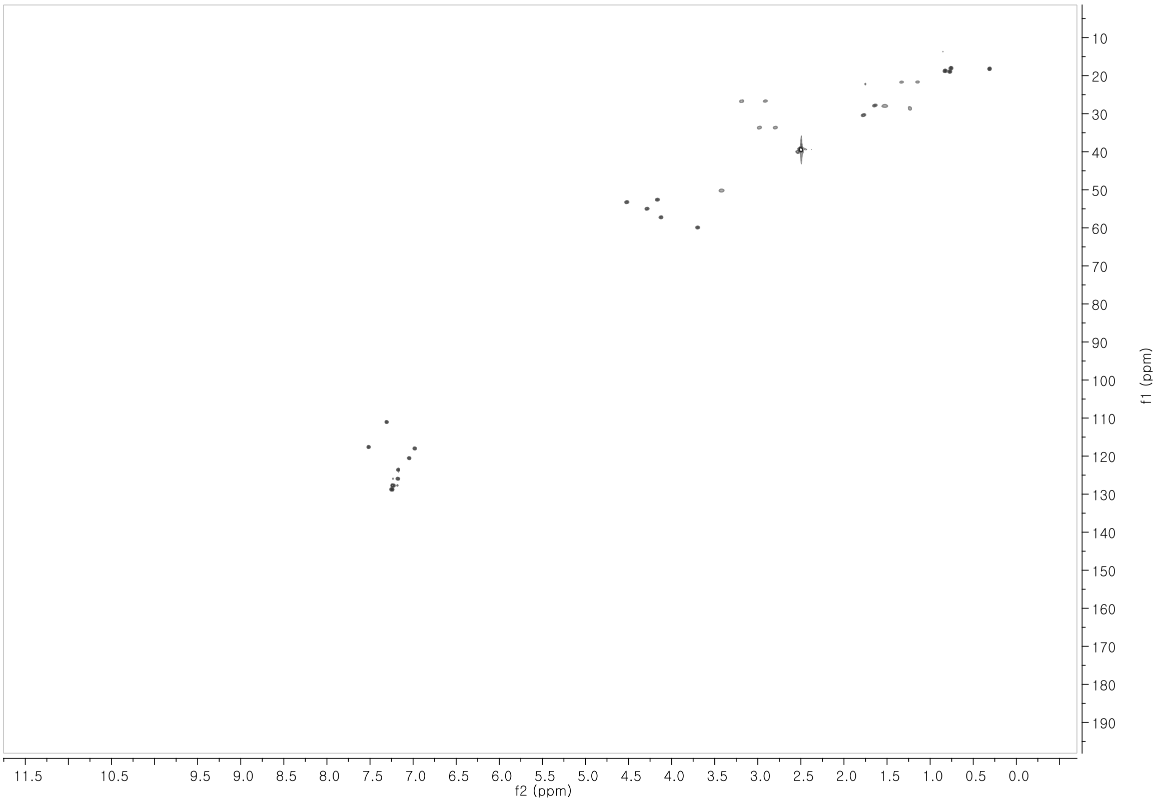

Figure S10. HMBC NMR spectrum (800 MHz) of pentaminomycin D (**2**) in DMSO-d_6.

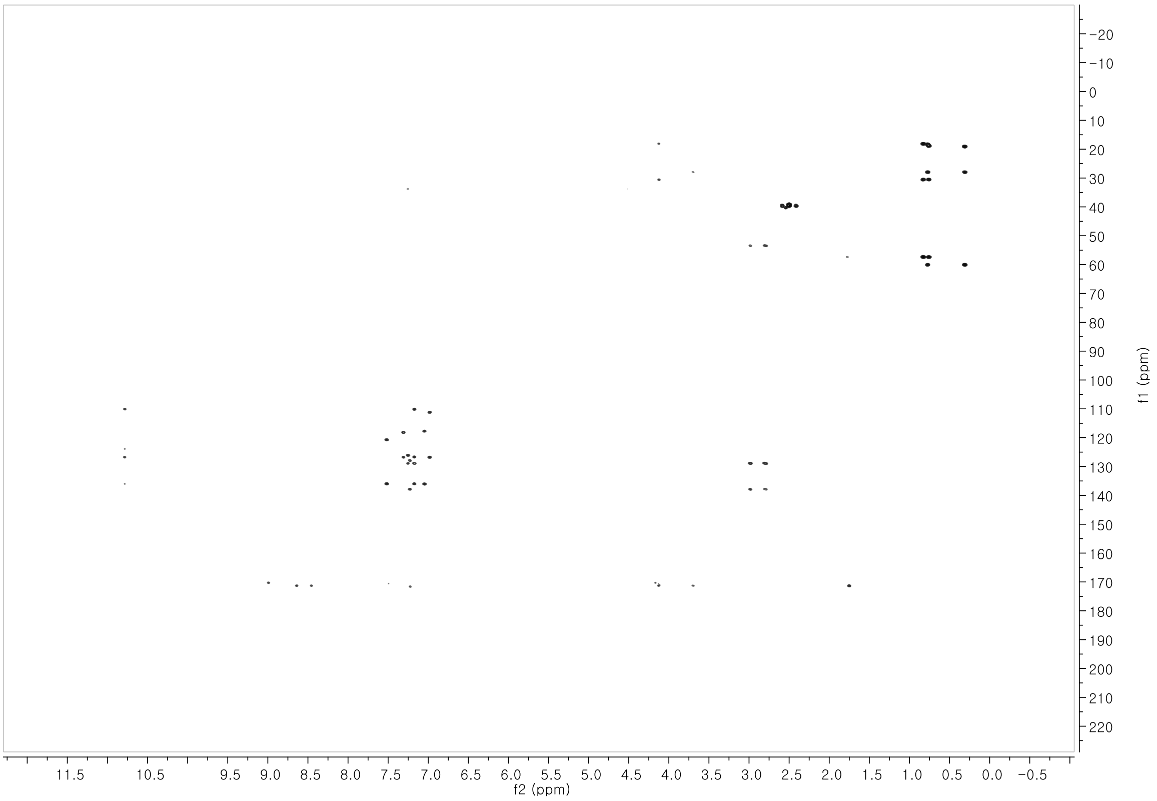

Figure S11. ^{1}H NMR spectrum (800 MHz) of pentaminomycin E (**3**) in DMSO-*d*$_6$.

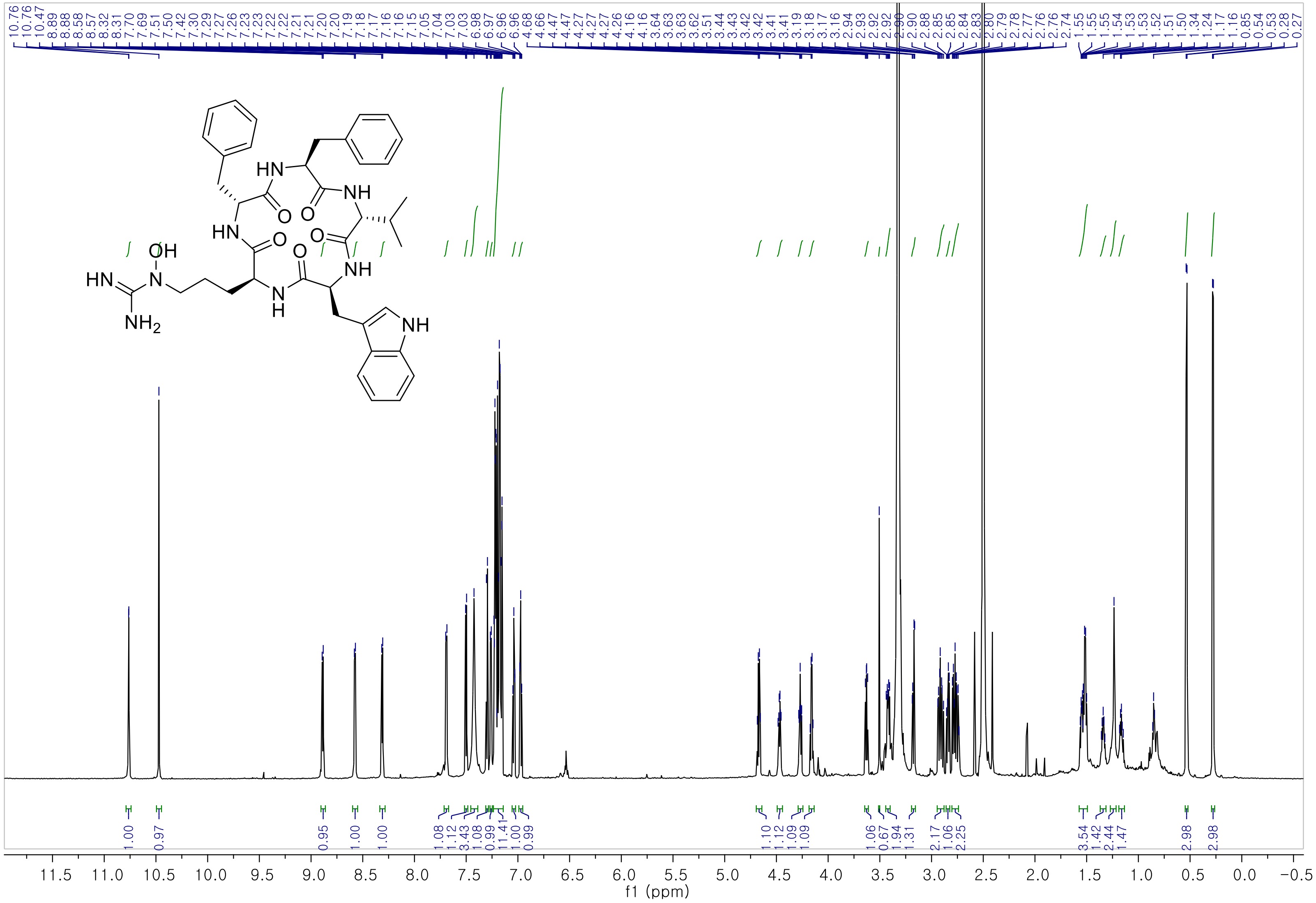

Figure S12. ^{13}C NMR spectrum (200 MHz) of pentaminomycin E (**3**) in DMSO-d_6.

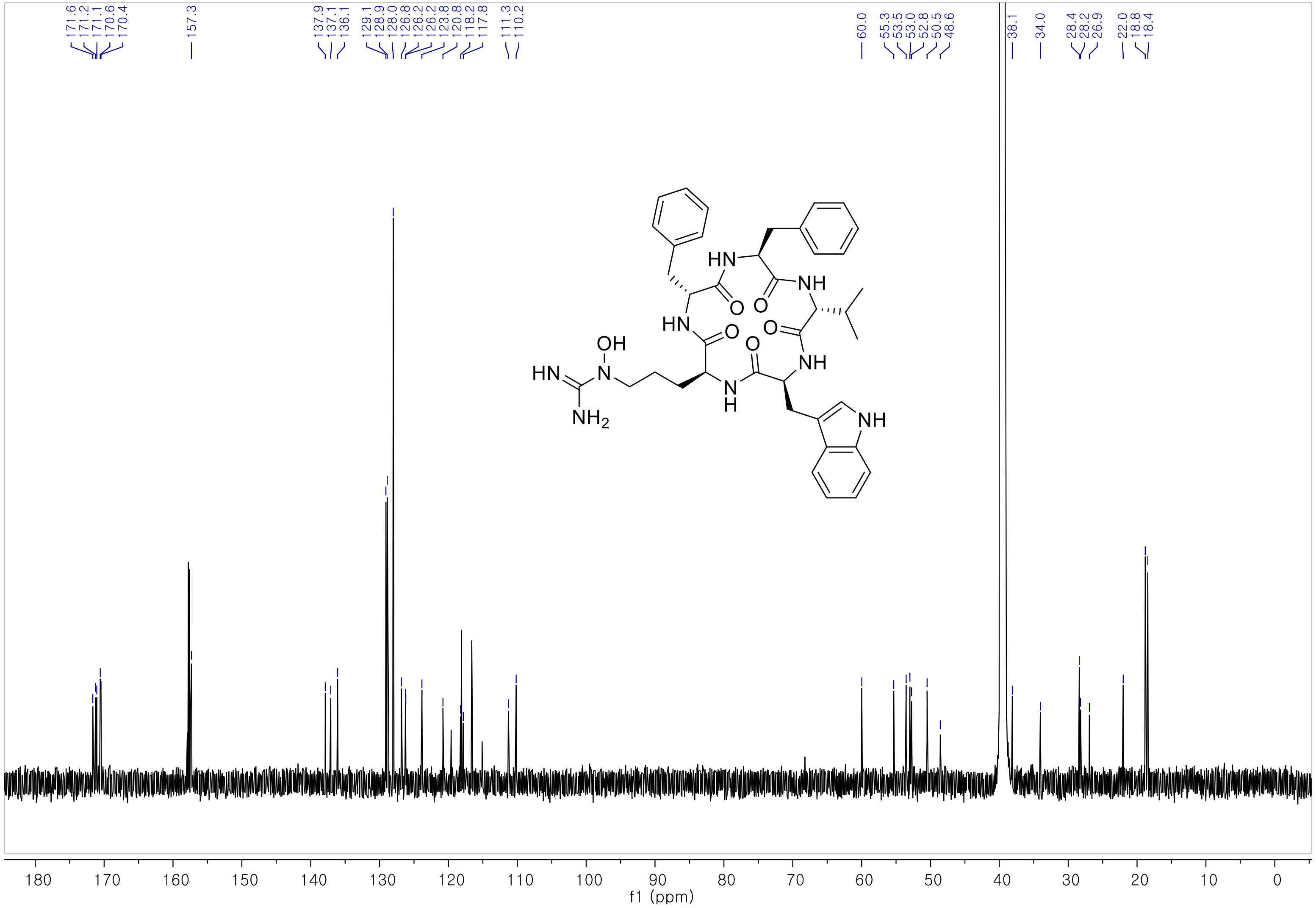

Figure S13. COSY NMR spectrum (800 MHz) of pentaminomycin E (**3**) in DMSO-d_6.

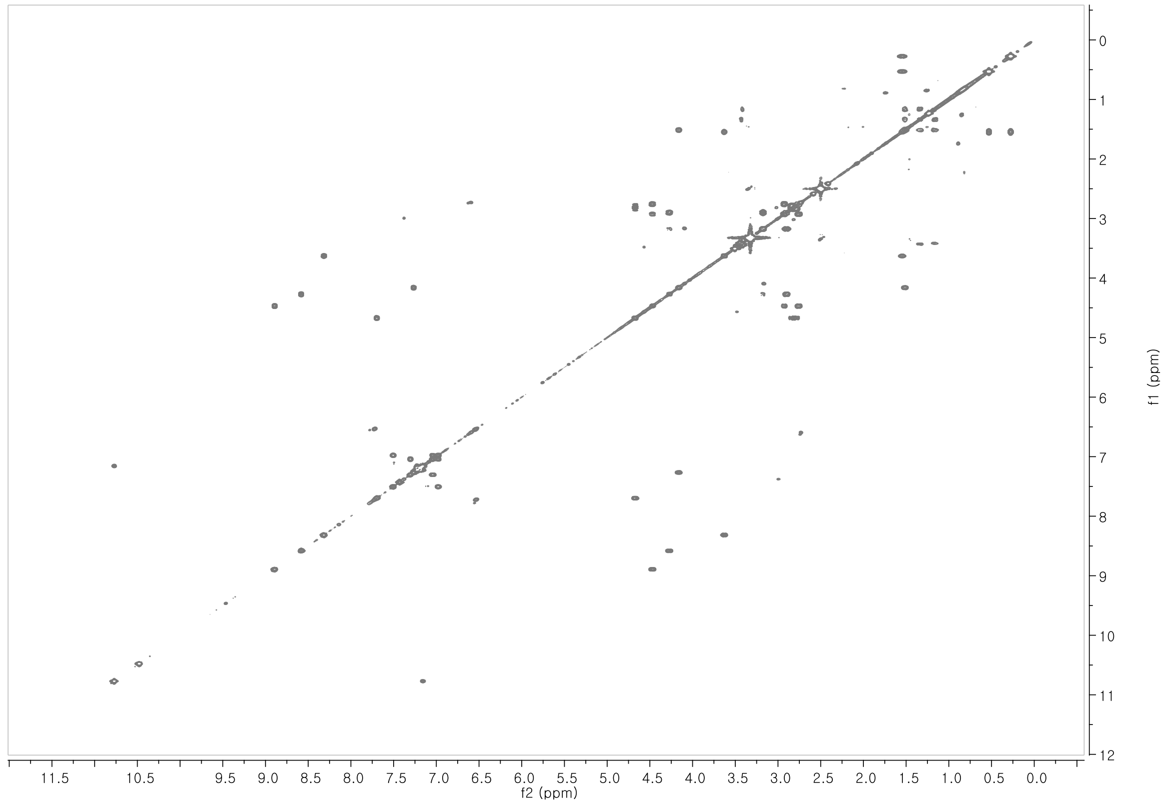

Figure S14. HSQC NMR spectrum (800 MHz) of pentaminomycin E (**3**) in DMSO-d_6.

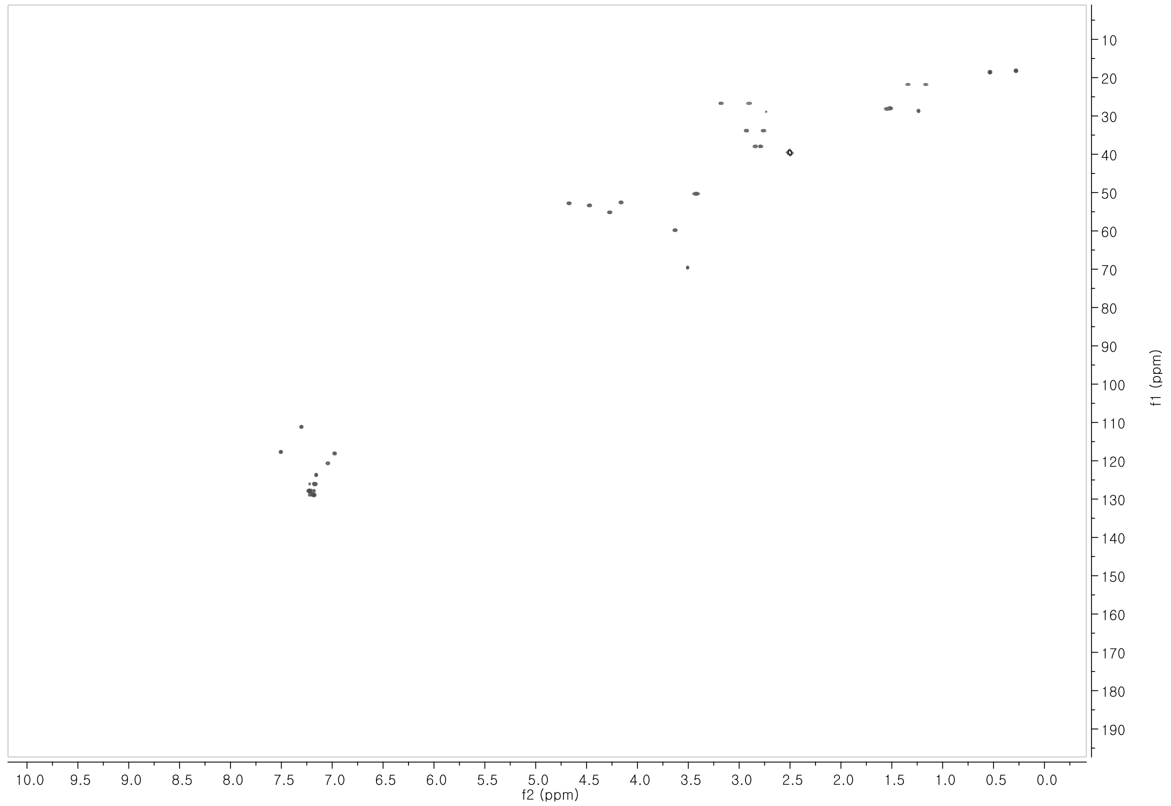

Figure S15. HMBC NMR spectrum (800 MHz) of pentaminomycin E (**3**) in DMSO-d_6.

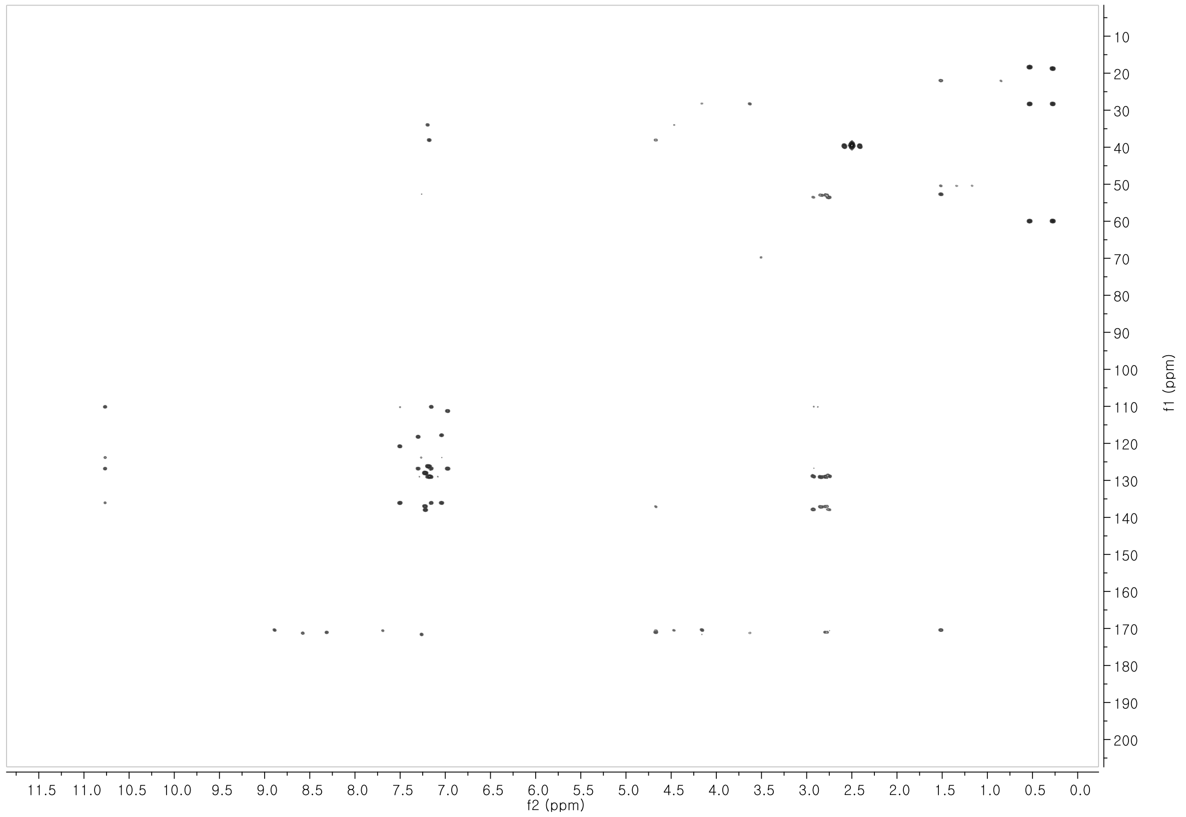

Figure S16. Production of BE-18257A and B detected by LC/MS analysis.

(a) Extract ion of BE-18257A from LC/MS profile of GG23 strain.

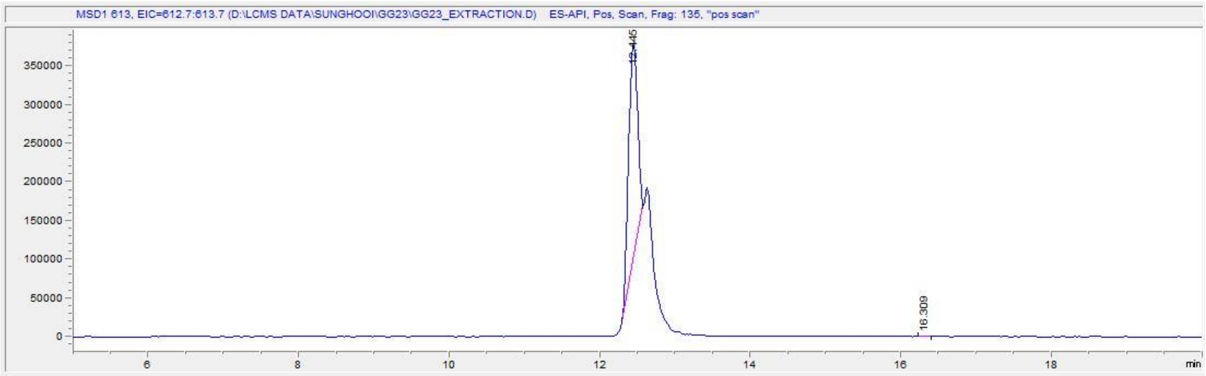

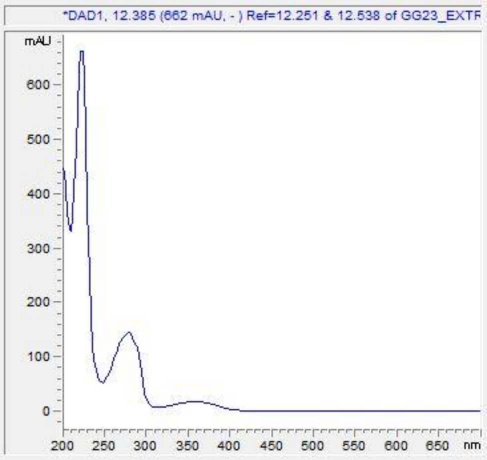

UV spectrum of BE-18257A

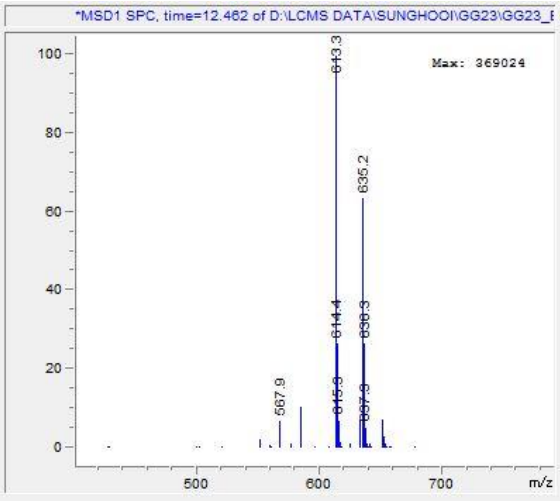

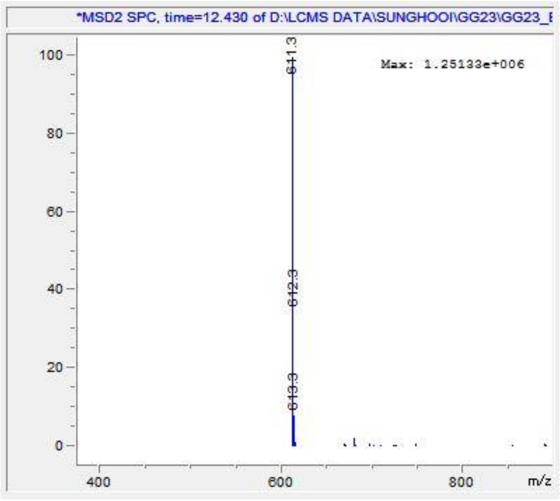

Positive (left) and negative (right) mass spectra of BE-18257A

(b) Extract ion of BE-18257B from LC/MS profile of GG23 strain.

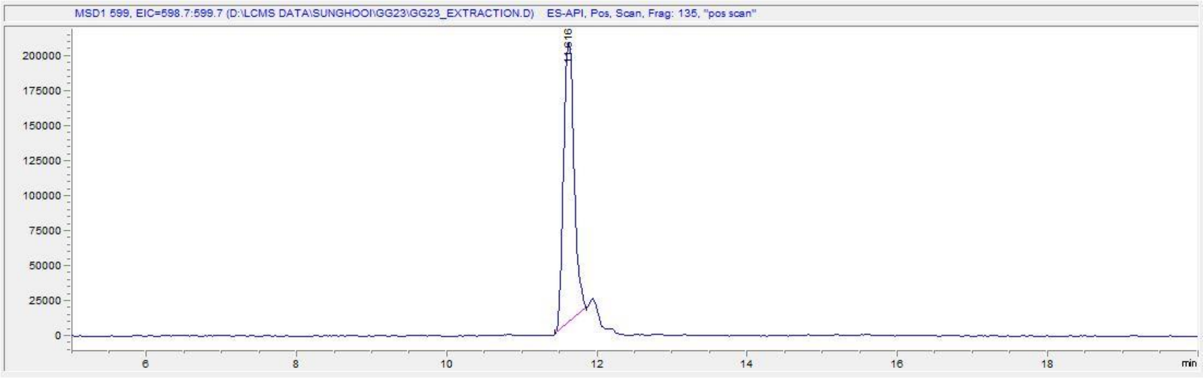

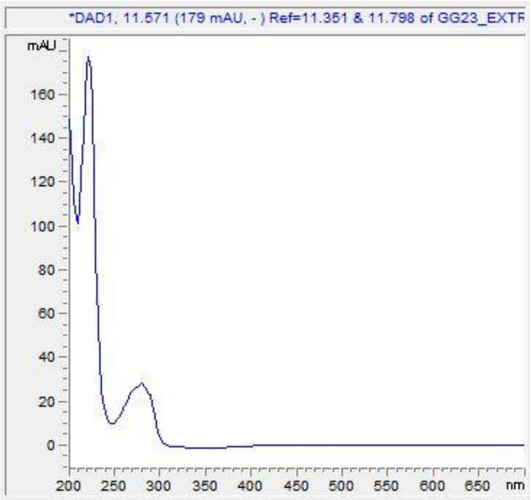

UV spectrum of BE-18257B

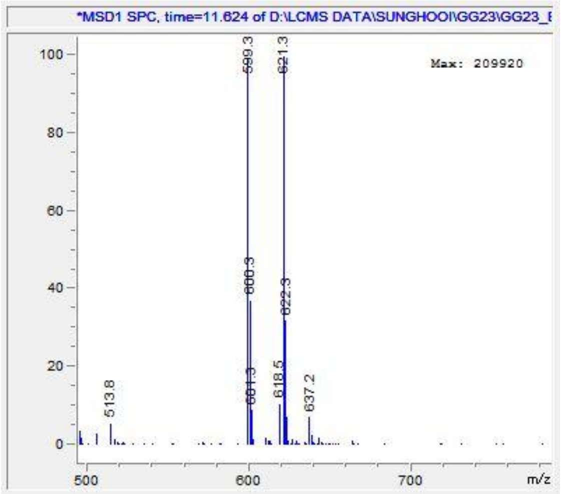

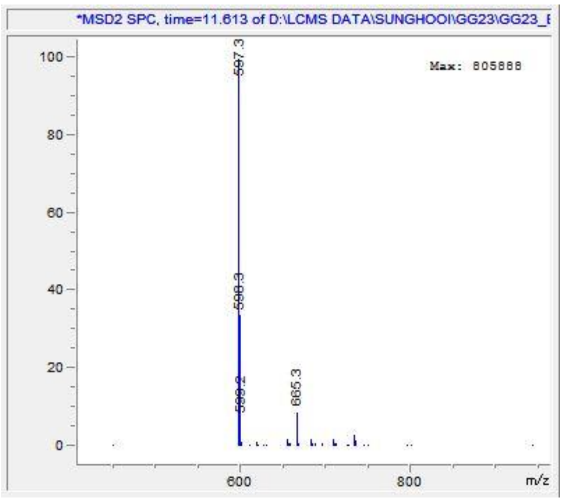

Positive (left) and negative (right) mass spectra of BE-18257B

Table S1. LC/MS analysis of L-FDLA derivatives of pentamnomycins D and E (**2**-**3**).

**Pentaminomycin D**				
**Amino acids**	Val	Arg	Trp	Phe
**$[M + H]^+$ (m/z)**	412	469	499	460
**Retention time (min)**				
**Reaction product**	37.5/44.6	22.6	41.0	47.5
**Authentic L with FDLA**	37.3	22.1	40.2	41.2
**Authentic D with FDLA**	43.8	21.4	43.9	46.0
**Supposed configuration**	**L** and **D**	**L from co-injection**	**L**	**D**

**Pentaminomycin E**				
**Amino acids**	Val	Arg	Trp	Phe
**$[M + H]^+$ (m/z)**	412	469	499	460
**Retention time (min)**				
**Reaction product**	43.5	21.7	39.8	40.4/45.9
**Authentic L with FDLA**	37.3	22.1	40.2	41.2
**Authentic D with FDLA**	43.8	21.4	43.9	46.0
**Supposed configuration**	**D**	**L from co-injection**	**L**	**L** and **D**

Figure S17. LC/MS chromatograms of Marfey's products of **2** and **3**.

(a) Valine

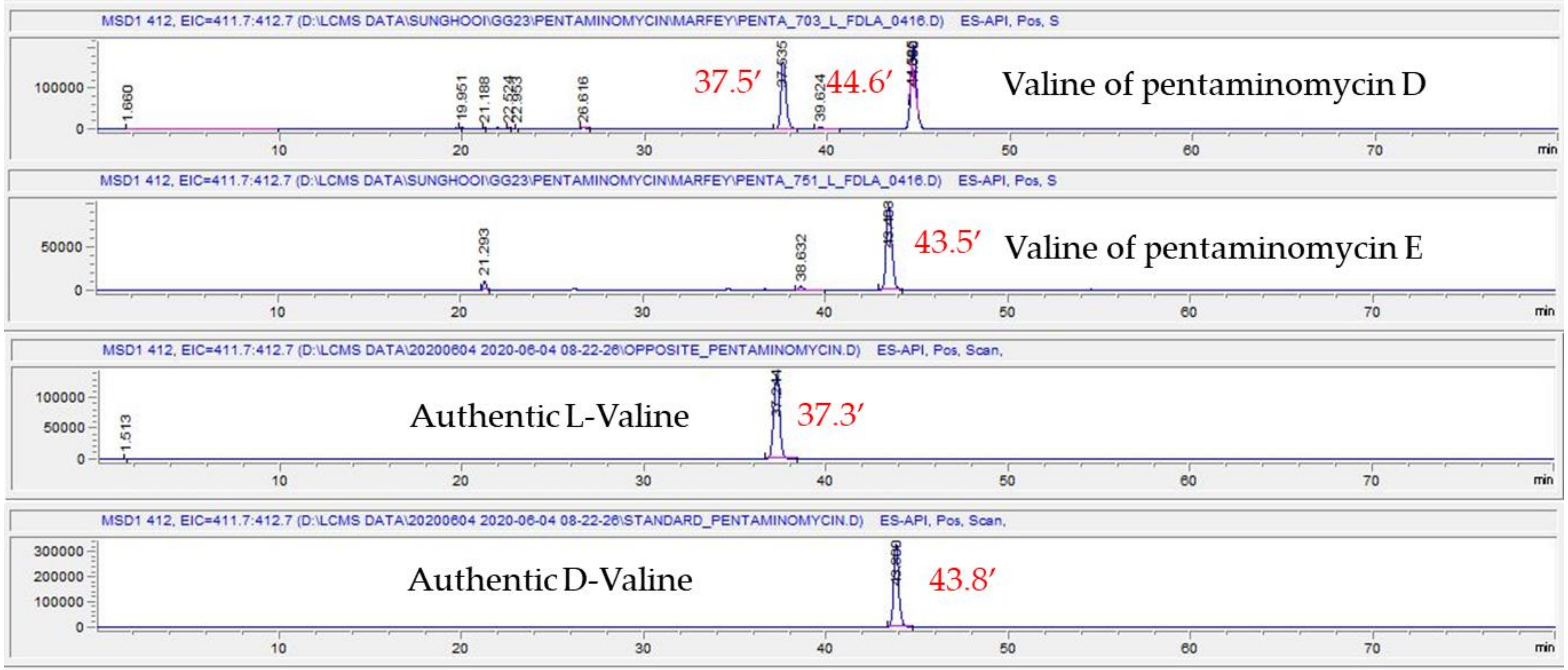

(b) Co-injection analysis of arginine

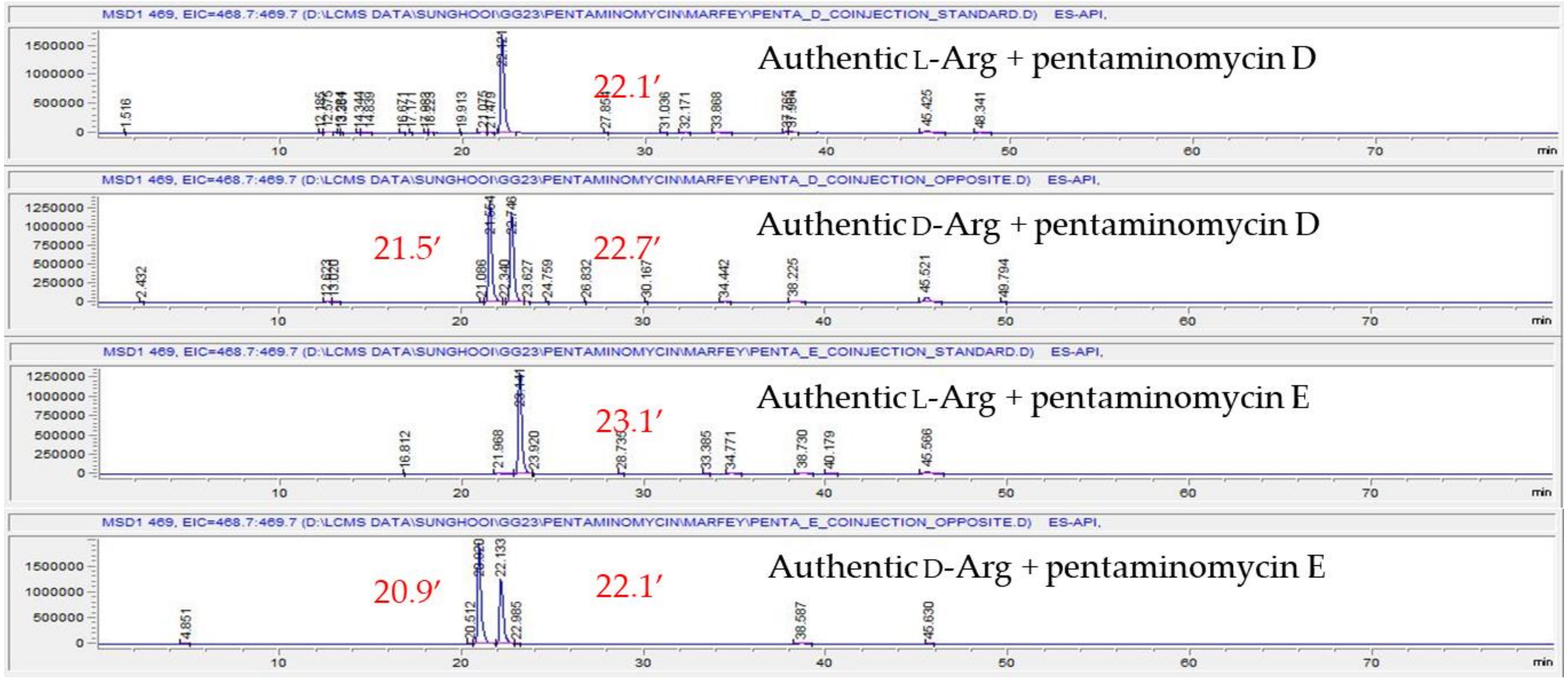

(c) Tryptophan

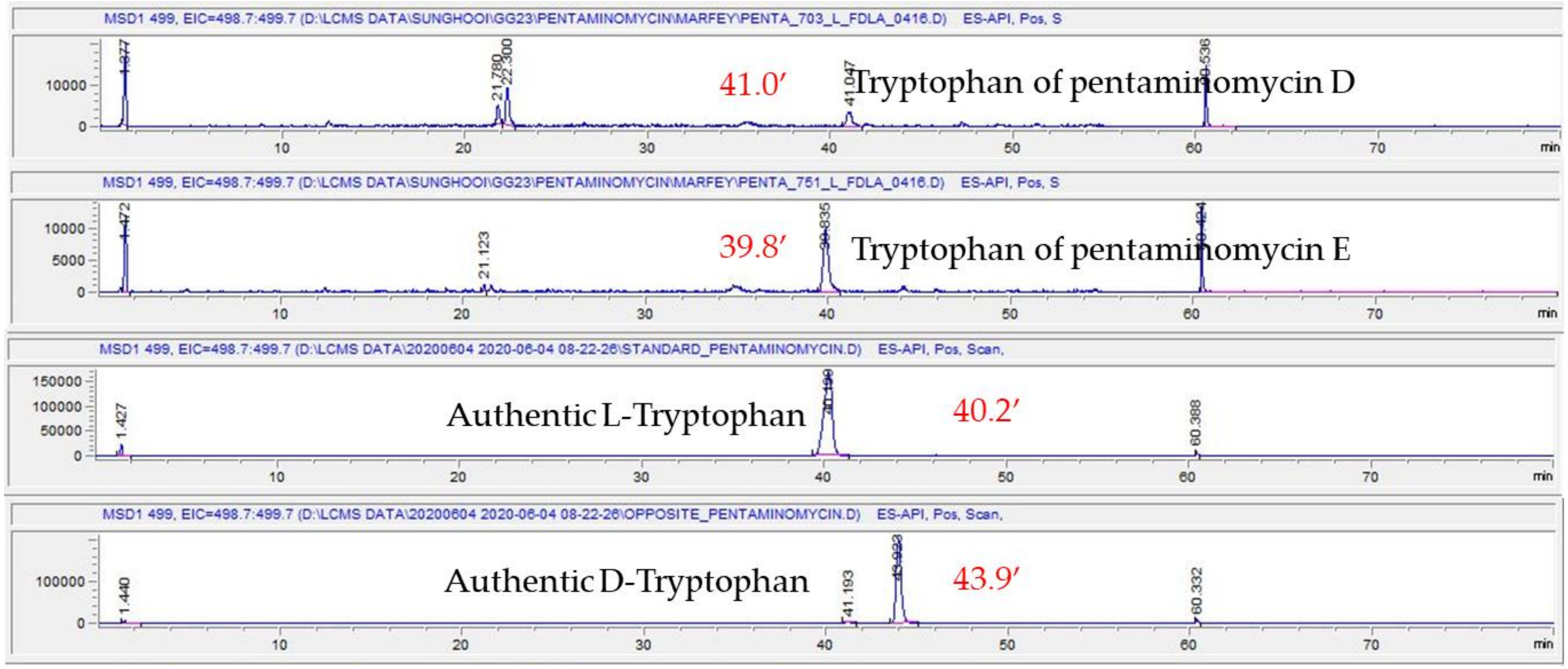

(d) Phenylalanine

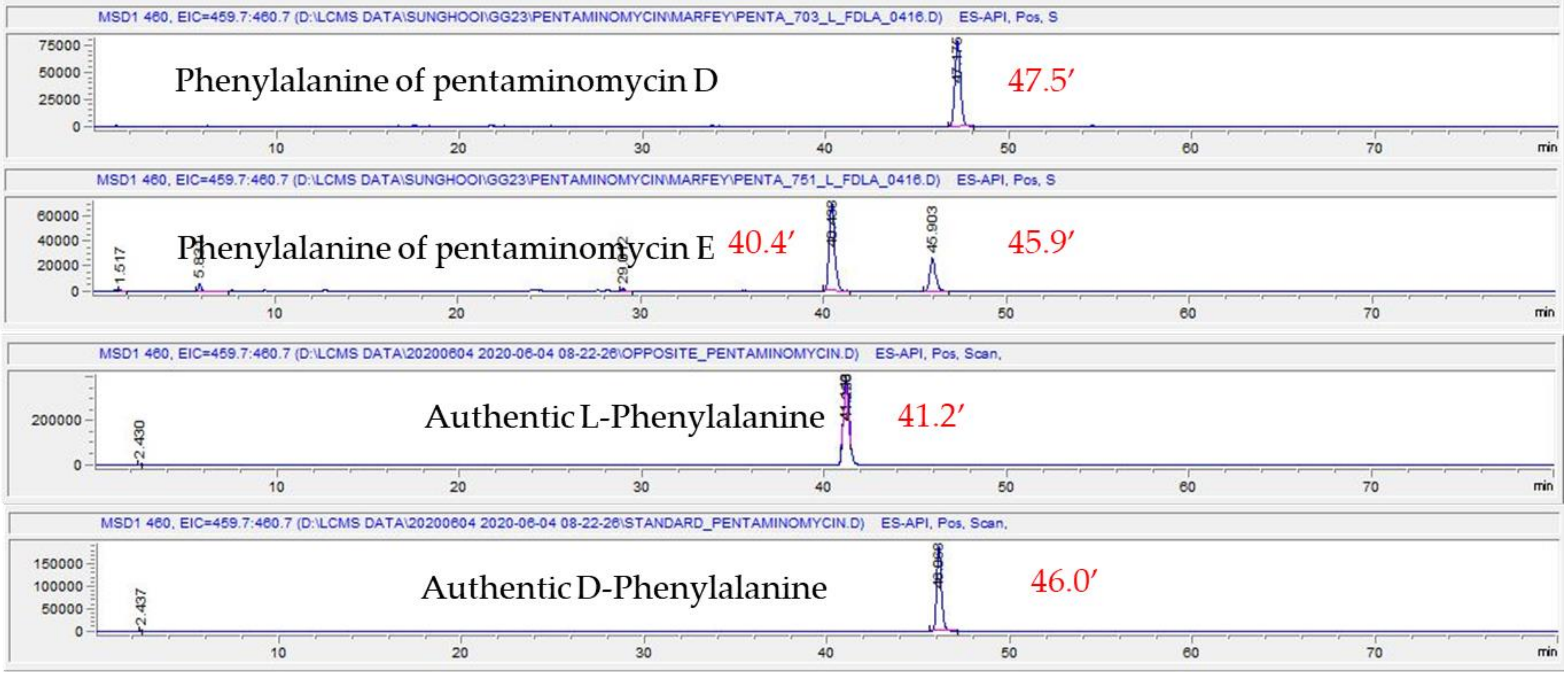

Table S2. AntiSMASH output table of *Streptomyces* sp. GG23.

Cluster	Type	From	To	Most similar known cluster
Cluster 1	Trans AT-PKS-like, trans AT-PKS, NRPS	1	98574	Phthoxazolin biosynthetic gene cluster (18% of genes show similarity)
Cluster 2	T1PKS-NRPS	167517	259556	Coelibactin biosynthetic gene cluster (36% of genes show similarity)
Cluster 3	T1PKS	267455	312805	Cyphomycin biosynthetic gene cluster (4% of genes show similarity)
Cluster 4	CDPS	326691	345329	Naseseazine C / C3-aryl pyrroloindolines biosynthetic gene cluster (66% of genes show similarity)
Cluster 5	NRPS	526279	576107	Actinonin biosynthetic gene cluster (100% of genes show similarity)
Cluster 6	Terpene	704748	726967	Hopene biosynthetic gene cluster (69% of genes show similarity)
Cluster 7	Betalactone	795864	824020	
Cluster 8	NRPS	896110	1042013	Surugamide A / D biosynthetic gene cluster (57% of genes show similarity)
Cluster 9	Terpene	1108589	1127449	Meoabyssomicin / abyssomicin biosynthetic gene cluster (6% of genes show similarity)
Cluster 10	T1PKS-NRPS	1215222	1322528	Kanamycin biosynthetic gene cluster (61% of genes show similarity)
Cluster 11	Bacteriocin	1493069	1504001	
Cluster 12	NRPS	1705219	1771853	Herboxidiene biosynthetic gene cluster (2% of genes show similarity)
Cluster 13	Siderophore	1906802	1920869	Ficellomycin biosynthetic gene cluster (3% of genes show similarity)
Cluster 14	NRPS-like	2783017	2824131	CDA1b / 2a / 2b / 3a / 3b / 4a / 4b biosynthetic gene cluster (7% of genes show similarity)
Cluster 15	Ectoine	2952161	2961869	Kosinostatin biosynthetic gene cluster (13% of genes show similarity)
Cluster 16	T2PKS-NRPS	2964906	3051850	Murayaquinone biosynthetic gene cluster (12% of genes show similarity)
Cluster 17	Nucleoside	3689480	3711978	Puromycin biosynthetic gene cluster (87% of genes show similarity)
Cluster 18	NRPS-like	5086755	5128865	Bottromycin A2 biosynthetic gene cluster (45% of genes show similarity)
Cluster 19	NRPS	5269602	5353193	Ulleungmycin biosynthetic gene cluster (25% of genes show similarity) → Pentaminomycins biosynthetic gene cluster
Cluster 20	Terpene	5805273	5826642	Daptomycin biosynthetic gene cluster (3% of genes show similarity)
Cluster 21	Lanthipeptide	6244105	6267284	
Cluster 22	Ectoine	6408859	6419263	Ectoine biosynthetic gene cluster (100% of genes show similarity)
Cluster 23	Siderophore	6478686	6490491	Desferrioxamine E biosynthetic gene cluster (100% of genes show similarity)
Cluster 24	NRPS	6686742	6750902	Telomycin biosynthetic gene cluster (11% of genes show similarity)
Cluster 25	CDPS	7148538	7167168	Naseseazine C / C3-aryl pyrroloindolines biosynthetic gene cluster (100% of genes show similarity)
Cluster 26	Terpene	7241740	7271334	Carotenoid biosynthetic gene cluster (54% of genes show similarity)
Cluster 27	NRPS	7632056	7692782	Dechlorocuracomycin biosynthetic gene cluster (16% of genes show similarity)
Cluster 28	NRPS	7766873	7825039	Vazabitide A biosynthetic gene cluster (8% of genes show similarity)
Cluster 29	CDPS, T1PKS-NRPS like	7859883	8014532	Levorin A3 / C06690 / RF-088-III / Candicidin A / UNII-AP5PF5W7U biosynthetic gene cluster (71% of genes show similarity)
Cluster 30	Lassopeptide	8263758	8286230	Incednine biosynthetic gene cluster (2% of genes show similarity)
Cluster 31	NRPS-like, T1PKS, T3PKS, NRPS, betalactone	8339738	8480684	Totopotensamide A / B biosynthetic gene cluster (64% of genes show similarity)

Table S3. Putative functions of ORFs in the pentaminomycin biosynthetic gene cluster. The pen cluster was deposited in GenBank under the accession number MT033037.

ORF	Size (aa)	Putative function	Best match	
			Organism / GenBank	Identity(%)/Similarity(%) (residues)
orf1	273	Carbon-nitrogen hydrolase	*Streptomyces* sp. NRRL F-5053 / WP_030896090.1	99 (272) / 99 (272)
orf2	332	Agmatinase	*Streptomyces* / WP_030891001.1	100 (332) / 100 (332)
penR1	355	Helix-turn-helix domain-containing protein	*Streptomyces* / WP_030896088.1	100 (355) / 100 (355)
orf3	55	hypothetical protein	*Streptomyces cacaoi* subsp. *cacaoi* / GEB53778.1	100 (55) / 100 (55)
orf4	388	Histidine kinase	*Streptomyces* / WP_030891007.1	100 (388) / 100 (388)
penR2	217	Response regulator transcription factor	*Streptomyces* / WP_030891008.1	100 (217) /100 (217)
penR3	203	Response regulator transcription factor	*Streptomyces* / WP_030891011.1	100 (203) / 100 (203)
orf5	424	hypothetical protein	*Streptomyces cacaoi* / WP_086815217.1	99 (420) / 99 (422)
orf6	75	hypothetical protein	unclassified *Streptomyces* / WP_030891014.1	100 (75) / 100 (75)
orf7	269	alpha/beta fold hydrolase	*Streptomyces* / WP_030891016.1	100 (269) / 100 (269)
orf8	128	hypothetical protein	*Streptomyces* sp. NHF165 / WP_159784838.1	99 (127) / 99 (127)
penR4	280	XRE family transcriptional regulator	*Streptomyces* sp. NHF165 / QHF93408.1	100 (280) / 100 (280)
orf9	72	DUF397 domain-containing protein	*Streptomyces* / WP_086815215.1	99 (71) / 98 (71)
orf10	78	hypothetical protein SCA03_63410	*Streptomyces cacaoi* subsp. *cacaoi* / GEB53790.1	97 (76) / 97 (76)
orf11	179	GNAT family N-acetyltransferase	*Streptomyces* sp. SB3404 / WP_165296511.1	83 (148) / 89(178)
penR5	82	MbtH family protein	*Streptomyces* / WP_030891033.1	100 (82) / 100 (82)
orf12	419	Histidine kinase	*Streptomyces cacaoi* subsp. *cacaoi* / GEB53794.1	100 (419) / 100 (419)

orf13	851	FtsX-like permease family protein	*Streptomyces* / WP_037853324.1	100 (851) / 100 (851)
orf14	238	ABC transporter ATP-binding protein	*Streptomyces* / WP_030896078.1	100 (238) / 100 (238)
penA	476	serine hydrolase	*Streptomyces cacaoi* / WP_086815212.1	99 (475) / 99 (475)
penN1	6188	NRPS (A-PCP-C-A-PCP-E-C-A-PCP-E-C-A-PCP-C-A-PCP-E)		
orf15	165	DUF2975 domain-containing protein	*Streptomyces* / WP_030891799.1	100 (165) / 100 (165)
orf16	96	Helix-turn-helix domain-containing protein	unclassified *Streptomyces* sp. / WP_078874108.1	90 (95) / 91 (96)
penR6	401	sensor histidine kinase	*Streptomyces* / WP_051857187.1	99 (394) / 100 (395)
orf17	280	hypothetical protein SCA03_67000	*Streptomyces cacaoi* subsp. *cacaoi* / GEB54149.1	100 (280) / 100 (280)
orf18	131	DUF742 domain-containing protein	*Streptomyces* / WP_030891786.1	100 (131) / 100 (131)
orf19	186	ATP-binding protein	*Streptomyces* / WP_030891784.1	100 (186) / 100 (186)
penB	451	Cytochrome P450	*Streptomyces* / WP_030891781.1	99 (450) / 100 (451)
penC	416	Cytochrome P450	*Streptomyces cacaoi* / WP_086815207.1	99 (415) / 99 (415)
orf20	271	3-hydroxybutyryl-CoA dehydratase	*Streptomyces cacaoi* subsp. *cacaoi* / GEB54144.1	99 (268) / 98 (268)
orf21	141	hypothetical protein	*Streptomyces* / WP_141275837.1	99 (140) / 100 (141)
penN2	5919	NRPS (A-PCP-C-A-PCP-E-C-A-PCP-C-A-PCP-C-A-PCP-E)		
orf22	69	hypothetical protein	*Streptomyces* / WP_030890086.1	100 (69) / 100 (69)
orf23	175	hypothetical protein	unclassified *Streptomyces* sp. / WP_030890089.1	100 (175) / 100 (175)
penR7	280	Helix-turn-helix domain-containing protein	*Streptomyces* / WP_030890094.1	100 (280) / 100 (280)
orf24	83	hypothetical protein	*Streptomyces* sp. NHF165 / WP_159784856.1	100 (83) / 100 (83)
orf25	252	DUF4328 domain-containing protein	unclassified *Streptomyces* sp. / WP_030890097.1	100 (217) / 100 (217)
orf26	79	hypothetical protein	*Streptomyces cacaoi* / WP_149564430.1	100 (70) / 100 (70)
orf27	120	hypothetical protein	*Streptomyces* sp. NRRL S-1868 / WP_030890101.1	100 (120) / 100 (120)

orf28	425	hypothetical protein	*Streptomyces* sp. NRRL S-1868 / WP_037867053.1	100 (425) / 100 (425)
orf29	104	4a-hydroxytetrahydrobiopterin dehydratase	*Streptomyces* / WP_078873989.1	99 (103) / 100 (104)
orf30	191	ATP-binding cassette domain-containing protein	*Streptomyces* / WP_030890110.1	100 (191) / 100 (191)
penR8	313	winged helix-turn-helix transcriptional regulator	*Streptomyces* / WP_063764732.1	100 (313) / 100 (313)
orf31	227	YqcI/YcgG family protein	*Streptomyces* sp. NRRL S-1868 / WP_078873990.1	100 (227) / 100 (227)
penD	583	anthranilate synthase component I	*Streptomyces* sp. NRRL F-5053 / WP_078875176.1	99 (577) / 99 (579)
penE	197	aminodeoxychorismate/anthranilate synthase component II	*Streptomyces* / WP_030890121.1	100 (197) / 100 (197)
penF	357	anthranilate phosphoribosyltransferase 2	*Streptomyces cacaoi* subsp. *cacaoi* / GEB51016.1	99 (356) / 99 (356)
penG	262	indole-3-glycerol phosphate synthase TrpC	*Streptomyces* sp. NRRL S-1868 / WP_030890127.1	100 (262) / 100 (262)
penH	488	3-deoxy-7-phosphoheptulonate synthase class II	*Streptomyces* / WP_051857087.1	100 (468) / 100 (468)
orf32	325	daunorubicin resistance protein DrrA family ABC transporter ATP-binding protein	*Streptomyces* / WP_030890133.1	100 (325) / 100 (325)
orf33	284	ABC transporter permease	*Streptomyces* / WP_078873992.1	100 (284) / 100 (284)
orf34	585	ubiquinol-cytochrome c reductase cytochrome b subunit	*Streptomyces* / WP_141275487.1	100 (585) / 100 (585)
orf35	129	cytochrome c oxidase subunit 4	*Streptomyces* / WP_030890141.1	100 (129) / 100 (129)
